# Sperm DNA fragmentation testing: Summary evidence and clinical practice recommendations

**DOI:** 10.1111/and.13874

**Published:** 2020-10-27

**Authors:** Sandro C. Esteves, Armand Zini, Robert Matthew Coward, Donald P. Evenson, Jaime Gosálvez, Sheena E. M. Lewis, Rakesh Sharma, Peter Humaidan

**Affiliations:** ^1^ ANDROFERT, Andrology and Human Reproduction Clinic Referral Center for Male Reproduction Campinas SP Brazil; ^2^ Department of Surgery (Division of Urology) University of Campinas (UNICAMP) Campinas SP Brazil; ^3^ Faculty of Health Aarhus University Aarhus Denmark; ^4^ Division of Urology Department of Surgery St. Mary's Hospital McGill University Montreal Québec Canada; ^5^ Department of Urology University of North Carolina Chapel Hill NC USA; ^6^ UNC Fertility Raleigh NC USA; ^7^ SCSA Diagnostics Brookings SD USA; ^8^ Sanford Medical School University of South Dakota Sioux Falls SD USA; ^9^ Unit of Genetics Department of Biology Universidad Autónoma de Madrid Madrid Spain; ^10^ Queens University Belfast Belfast UK; ^11^ Examenlab Ltd. Belfast UK; ^12^ American Center for Reproductive Medicine Cleveland Clinic Cleveland OH USA; ^13^ Fertility Clinic Skive Skive Regional Hospital Skive Denmark

**Keywords:** assisted reproductive technology, male infertility, practice guideline, semen analysis, sperm DNA fragmentation

## Abstract

We herein summarise the evidence concerning the impact of sperm DNA fragmentation in various clinical infertility scenarios and the advances on sperm DNA fragmentation tests. The collected evidence was used to formulate 41 recommendations. Of these, 13 recommendations concern technical aspects of sperm DNA fragmentation testing, including pre‐analytical information, clinical thresholds and interpretation of results. The remaining 28 recommendations relate to indications for sperm DNA fragmentation testing and clinical management. Clinical scenarios like varicocele, unexplained infertility, idiopathic infertility, recurrent pregnancy loss, intrauterine insemination, in vitro fertilisation/intracytoplasmic sperm injection, fertility counselling for men with infertility risk factors and sperm cryopreservation have been contemplated. The bulk evidence supporting the recommendations has increased in recent years, but it is still of moderate to low quality. This guideline provides clinicians with advice on best practices in sperm DNA fragmentation testing. Also, recommendations are provided on possible management strategies to overcome infertility related to sperm DNA fragmentation, based on the best available evidence. Lastly, we identified gaps in knowledge and opportunities for research and elaborated a list of recommendations to stimulate further investigation.

## 1 BACKGROUND

### 1.1 Overview of male infertility

Infertility affects over 180 million people worldwide. The male factor is found in nearly 10% of all couples and is responsible for about 50% of infertility cases (Agarwal et al., 2015). Male infertility, in particular, is a disorder of the reproductive system, caused primarily by male factors involving deficiencies in the semen, genetic and congenital conditions, anatomical defects, endocrine disturbances, immunological or functional abnormalities, sexual conditions incompatible with intercourse and chronic illness (Zegers‐Hochschild et al., 2017).

The incidence of male infertility has apparently increased in parallel with decay in semen quality (Andersson et al., 2008; Evenson et al., 1999; Swan et al., 2000). Male factor infertility adversely affects reproductive outcomes even under assisted reproductive technology (ART) settings (Boulet et al., 2015; Nangia et al., 2011). Despite this fact, the male partner is often neglected during the evaluation and treatment of infertility (Petok, 2015). A critical aspect relates to the fact that the cause of male infertility remains unexplained in up to 50% of patients using classic assessments, and ART treatments are widely available to successfully bypass the male factor in many cases (Esteves & Chan, 2015; Hamada et al., 2012; Jungwirth et al., 2015). Male infertility is vast and complex, covering a broad spectrum, including conventional and novel diagnostic methods, hormonal control, genetic and epigenetic regulation, interventional therapy and ART. The development of robust methods for male infertility diagnosis is urgently needed, since the routine semen analysis—the laboratory backbone of infertility investigation—has shown little progress over the years (Barratt et al., 2018).

### 1.2 Impact of sperm DNA fragmentation on fertility

Sperm DNA integrity is indispensable for the birth of healthy offspring (Krawetz, 2005). Increasing evidence indicates that sperm DNA fragmentation (SDF), a marker of damaged chromatin, has an independent and remarkable role in male infertility and reproductive success (Agarwal, Majzoub, et al., 2016; Aitken, 2016, 2017a; Bui et al., 2018; Esteves, Gosálvez, et al., 2015; Rima et al., 2016; Saleh et al., 2002; Sergerie et al., 2005).

Sperm DNA fragmentation may adversely impact sperm fertilising potential, particularly when DNA damage levels are high (González‐Marín et al., 2012; Lopes et al., 1998; Simon et al., 2010, 2011). Levels of oxidative stress that are not sufficient to induce cell death via apoptosis can disrupt all sperm function aspects, including motility, sperm–zona recognition, acrosomal exocytosis and sperm–oocyte fusion (Aitken, 2020). However, spermatozoa with damaged chromatin may retain their fertilising ability (Zenzes et al., 1999). The mixed results obtained in studies evaluating SDF and fertilisation capacity could be explained, at least in part, by the diverse nature of the DNA damage and the oocyte's repair capacity.

Indeed, the impact of SDF on reproductive success will depend on the balance between the extent of DNA damage and the oocyte's DNA repair capacity (Champroux et al., 2016; Menezo et al., 2007). While the repair process probably occurs at the pronuclei stage before syngamy, it has been postulated that sperm DNA damage exceeding the oocyte's repair capacity—or oocyte's failure to repair DNA damage—influences the embryo development potential and the health of the offspring (Martin et al. 2019; Horta et al., 2020). In such cases, protaminised sperm chromatin cannot be adequately replaced by histones needed for normal DNA replication (Gosálvez, Fernández, et al., 2015). For example, oxidative DNA lesions may lead to transversion mutations (e.g., G‐C to T‐A), altering gene expression if not repaired by the oocyte base excision repair (BER) enzymes before zygote S‐phase. As a result, the embryo may fail to develop or implant in the uterus or may be aborted naturally at a later stage. Conversely, if existing DNA repair mechanisms within the oocyte are able to restore a biologically stable genome, normal syngamy and subsequent embryonic development can occur.

Accordingly, it has been suggested that the impact of SDF on reproductive success would be better observed post‐fertilisation, and the effect will depend mainly on balance between the type and extent of sperm DNA damage and the oocyte's DNA repair capacity (Champroux et al., 2016; Menezo et al., 2007). SDF may not be perceived on fertilisation but rather causes a late paternal effect related to paternal gene expression in the 4‐ to 8‐cell embryo (Tesarik et al., 2004). Horta et al. (2020) recently demonstrated experimentally that despite high levels of induced SDF, IVF fertilisation may occur normally, and SDF can be corrected by oocytes from younger females, thus allowing for normal embryo development.

The most convincing evidence of an adverse effect of SDF on fertility comes from animal studies. In these studies, the relationship between SDF and natural or assisted reproduction outcomes is not influenced by confounding variables, as it is in clinical studies (Evenson et al., 1980; Li & Lloyd, 2020). Human IVF and ICSI models using proven fertile donor oocytes have also been utilised to study the impact of SDF on fertility (Gosálvez et al., 2013; Nuñez‐Calonge et al., 2012). An ICSI study using donor oocytes of proven fertility showed that SDF rates of nonpregnant couples (34.9%) were higher than that of pregnant couples (25.3%; *p* < 0.001; Gosálvez et al., 2013). Using a ROC curve and Youden index, the authors found that a threshold SDF value of 24.8% (by SCD assessed in the neat semen) yielded a 75% sensitivity and 69% specificity for pregnancy prediction. Additionally, a variety of human studies using different designs and endpoints have explored the relationship between SDF and fertility, including natural pregnancy, unexplained infertility, recurrent pregnancy loss (RPL), intrauterine insemination (IUI), in vitro fertilisation (IVF) and intracytoplasmic sperm injection (ICSI). In such studies, however, confounding factors (e.g. female age, presence of comorbidities) may influence the effect magnitude of SDF on reproductive success.

### 1.3 Association between sperm DNA fragmentation and male infertility

Infertile men frequently have high levels of SDF in neat semen. A 2018 systematic review and meta‐analysis, including over four thousand men and 27 studies, revealed that the standardized mean difference in SDF rates between infertile versus fertile men was 1.6% (95% confidence interval [CI]: 1.2–2.1; *p* < 0.001; Santi et al., 2018). In this report, infertile men were those with unexplained infertility (15 studies) or abnormal routine semen analysis (12 studies), whereas fertile counterparts were men with proven fertility (14 studies), healthy donors (8 studies), volunteers (2 studies) and men with normal routine semen analysis (4 studies). Accordingly, the SDF threshold level that most optimally discriminated infertile from fertile men was 20% (area under the curve [AUC]: 0.84; *p* < 0.001; sensitivity: 79%; specificity: 86%). Many conditions, including varicocele, chronic illness, accessory gland infections, advanced paternal age, lifestyle, obesity, occupational and environmental factors, medications, ionising and nonionising radiation and heat exposure, have been associated with elevated SDF levels (Esteves, 2019; Roque & Esteves, 2018). These conditions can promote SDF mainly by causing defective spermatogenesis, evoking abortive apoptosis or increasing the generation of reactive oxygen species (ROS). Excessive ROS represent a significant causative factor of SDF in live spermatozoa (Agarwal et al., 2019).

Our current understanding of the relationship between SDF and male infertility primarily relates to the comparisons among populations of fertile men (whose fertility may have changed since they last produced an offspring) and infertile men. While time to pregnancy (TTP) of less than 12 months from stopping contraception and ability to conceive should be ideally used as the criterion to classify fertile men (Buck Louis et al., 2014), this definition is not uniformly applied in all studies (Santi et al., 2018). Moreover, male infertility has many causes and, therefore, the studied population should be adequately characterised to assess the conditions possibly associated with excessive oxidative stress.

### 1.4 The rationale of sperm DNA fragmentation testing

Sperm DNA fragmentation testing has been used to attain more in‐depth knowledge about sperm quality due to the critical function of sperm DNA integrity for healthy embryonic development and successful reproductive outcome.

The rationale in performing SDF testing relates primarily to the adverse impact of defective sperm chromatin on reproductive success as a whole rather than sperm fertilising capacity in particular. Nonetheless, the predictive value of SDF as a single contributor to reproductive success is challenging because pregnancy is affected by a multitude of controlled and uncontrolled factors. Moreover, in routine clinical settings, male infertility is often a nonsingle factor condition, which may result from a series of nonexclusive and possibly inter‐related events including defective spermatogenesis during chromatin remodelling, oxidative stress, subclinical infections, presence of chromosomal abnormalities, constitutive genetic conditions, genomic modifications, such as telomere‐shortening and lifestyle/environmental stressors.

Thus, although SDF predictive values should be considered when interpreting test results, infertility is a couple's problem, and a single test of gamete dysfunction from just one partner is limited to predict treatment outcome. Nonetheless, the existing evidence indicates that the probability of a successful pregnancy outcome (natural and assisted) is influenced by the SDF level. Moreover, a growing body of evidence referenced in this manuscript supports the hypothesis that SDF is associated with various pre‐conception developmental impairments and also post‐conception issues such as miscarriages and increased susceptibility to progeny diseases.

Despite the robust association between SDF and infertility, the limited knowledge of SDF tests' characteristics and a common opinion that SDF is untreatable have prevented the broad application of testing in routine practice (Esteves, Agarwal, Cho, et al., 2017). Moreover, clear indications for SDF testing are limited; only recently, clinical practice recommendations on its use were proposed (Agarwal, Majzoub, et al., 2016; Atik et al., ; Salonia et al., 2020).

### 1.5 The need for clinical practice guidelines

The continuous expansion in medical information and the need to refine efficiency in diagnosing and treating clinical conditions have been the driving forces for the clinical practice guidelines (CPG's) role and utility. Currently, about eight guidelines on male infertility have been developed by expert panels from many societies (reviewed by Esteves & Chan, 2015; Roque & Esteves, 2016; Shridharani et al., 2016). A common trait among all guidelines is the scanty available evidence to elaborate recommendations. Most recommendations are graded ‘B’, ‘C’ or ‘D’, thus indicating that the evidence used to formulate recommendations originates overwhelmingly from nonrandomised studies and expert opinion.

With regard to SDF testing, societies like the American Urological Association (AUA) and the American Society for Reproductive Medicine (ASRM) have not recommended the use of SDF testing during the routine infertility evaluation mainly due to insufficient data and lack of effective treatment options to overcome infertility in such cases (Jarow et al., 2011; Practice Committee of the American Society for Reproductive Medicine, 2013, 2015). However, more recently, in 2015, the ASRM guidelines conceded that varicocele repair and antioxidant use might be of value to reduce SDF and that testing for SDF might be clinically informative for IUI, IVF and ICSI outcomes. It also acknowledged that spermatozoa retrieved from the testis of men with elevated SDF in the neat semen could have better DNA quality that ejaculated counterparts (Practice Committee of the American Society for Reproductive Medicine, 2015).

Recently, three CPG included specific recommendations concerning SDF testing (Agarwal et al., 2017; Bender Atik et al. 2018; Salonia et al., 2020). Briefly, the 2017 Society for Translational Medicine guideline included indications for testing (Agarwal et al., 2017). This guideline recommends testing for couples with (a) unexplained infertility, (b) recurrent pregnancy loss (RPL), (c) male patients with risk factors (e.g. inadequate lifestyle, exposure to toxicants), and (d) after failed unexplained IUI, IVF or ICSI. This CPG was the first of its kind to aggregate the available evidence and provide clinicians with guidance for management. Several experts critically analysed the document from many angles (see Esteves, Agarwal, Cho, et al., 2017; Majzoub, Agarwal, Cho, Esteves, 2017; Translational Andrology and Urology (Sperm DNA Fragmentation). The consensus was that the recommendations made were primarily based on low‐quality evidence, indicating that more research should be conducted.

The CPG on RPL by the European Society for Human Reproduction and Embryology (ESHRE) is a vast document that contains a subsection specifically addressing SDF testing (Bender Atik et al. 2018). It underscores that SDF testing in couples with RPL could be considered for explanatory purposes. The ESHRE guidelines concluded that there is evidence supporting an association between RPL and SDF, and this association seems to be independent of female factors. However, the guidelines pointed out that the impact of interventions to decrease SDF on RPL warrants further investigation.

Lastly, the 2020 European Association of Urology (EAU) guidelines on male infertility dedicated a few sections to SDF testing and the impact of SDF in varicocele and unexplained infertility (Salonia et al., 2020). The EAU guidelines recommend SDF testing in (a) couples with RPL following natural conception, IUI and IVF/ICSI, and (b) men with unexplained infertility. Moreover, it is suggested that in men with unexplained infertility and elevated SDF, who have experienced failed IUI, IVF, or ICSI, testicular sperm retrieval may be used for ICSI as a way to overcome infertility related to impaired sperm DNA quality. The EAU document underlines that in the latter, patients must balance the risks of undergoing an invasive procedure in an otherwise normozoospermic or unexplained condition. Besides, the EAU guidelines acknowledge the critical role SDF in the pathophysiology of infertility related to varicocele, and the potential benefit of varicocele repair to reduce SDF. A specific recommendation is given in this regard, which underscores that varicocele repair may be considered in men with elevated SDF and otherwise unexplained infertility or who have suffered from failed ART treatment, including RPL and implantation failure. It is implied, therefore, that SDF testing should be used to identify men who could benefit from varicocele repair.

Clinical practice guidelines are useful tools to help clinicians to refine the quality of health care provided to men with infertility. CPGs may also deter potentially wrongful or fruitless interventions during the evaluation and management of male infertility (Esteves & Chan, 2015). Since the publication of the guidelines mentioned above, more data have been made available, and new possible indications for SDF have emerged. Besides, new data unfolded the potential benefit of medical and surgical interventions to decrease SDF. Therefore, we reviewed the existing data on SDF testing indications in a diverse range of clinical scenarios and elaborated recommendations based on the best evidence and expert judgment.

## 2 GUIDELINE DEVELOPMENT

### 2.1 Guideline development group and evidence search

The current guideline was developed independently by the Sperm DNA Fragmentation Study Group (SFRAG). The coordinator (SCE) drafted the key questions and invited experts in the field, including reproductive urologists (AZ, RMC), scientists with well‐known expertise in the technical aspects of SDF tests (RS, DPE, SEML, JG) and one reproductive endocrinologist (PH). Based on defined keywords (Male infertility; Sperm DNA fragmentation; Spermatozoa; Human; Assisted Reproductive Technology; Intrauterine insemination; In vitro Fertilisation; Intracytoplasmic sperm injection; Varicocele; Recurrent pregnancy loss; Unexplained infertility; Idiopathic infertility; Lifestyle risk reduction; Male infertility factors), the literature search was performed in PubMed/MEDLINE from inception up to 31 May 2020.

### 2.2 Evidence summary

The coordinator prepared a summary of findings based on existing systematic reviews and meta‐analyses and controlled trials or relevant cohort studies and case reports when the former were not available. The guideline development group (GDG) discussed the summary evidence and provided additional supporting evidence if applicable, which served the basis for the draft recommendations.

### 2.3 Formulation of recommendations

The coordinator prepared the draft recommendations and discussed them with the GDG to reach an agreement on the final recommendations. For each recommendation, a strength rating based on GDG expert judgment and the grade of recommendation, according to the Oxford Centre for Evidence‐Based Medicine Levels of Evidence (OCEBM Levels of Evidence Working Group), was included. The strength rating was based on clinical expertise, taking into account the overall quality of evidence, the balance between risks and benefits, and the likely impact on patient preferences and values. We classified the strength of recommendations as strong or conditional. Strong recommendations imply that most individuals in that situation should receive testing or intervention. By contrast, conditional recommendations imply that various choices might be suitable for individual patients and that healthcare practitioners should help each patient reach a decision coherent with a patient‐centred approach.

## 3 WHAT ARE THE MECHANISMS CAUSING SPERM DNA DAMAGE?

In healthy spermatozoa, the chromatin is characterised by a linear disposition of the nucleotides along each DNA strand and the lack of both single and double DNA strand breaks, nucleotide modifications or base loss (Cortés‐Gutiérrez et al., 2014). The sperm chromatin has plenty of alkali‐labile sites, mainly localised in the repetitive DNA sequences, prone to DNA torsion during chromatin packing. Chromatin damage is an inclusive term that accounts for any defects in the DNA structure. These defects include (a) single or double DNA strand breaks, (b) base deletion or modification, (c) interstrand or intrastrand DNA cross‐linkage and (d) protamine deficiency and/or mispackage via defective DNA–protein cross‐link (reviewed by Esteves et al., 2014). It may occur during spermatogenesis, spermiogenesis, epididymal transit or post‐ejaculation. In particular, SDF relates to the breaks at the DNA strands, which are termed single‐strand (SS‐DBs) or double‐strand breaks (DS‐DBs). SS‐DBs give rise to free 5′–3′ ends affecting only one DNA strand, whereas its template remains undamaged. By contrast, DS‐DBs are characterised by blunt 5′–3′ ends affecting both DNA strands.

As mentioned above, SDF involves multiple causative factors, including varicocele, lifestyle‐related habits, exposure to occupational and environmental toxicants, ageing and infections (Agarwal, Majzoub, et al., 2016; Cho et al., 2016; Esteves, Santi, et al., 2020; Evenson et al., 2020). At the cellular level, these factors can promote DNA breaks through nonmutually exclusive mechanisms, namely, sperm chromatin maturation defects, apoptosis and OS (Esteves, et al., 2014; Gosálvez, López‐Fernández, et al., 2015).

### 3.1 Defects in chromatin compaction and DNA repair mechanisms

Transition proteins and protamines replace 85% of histones during spermiogenesis (Esteves, et al., 2014; Majzoub, Agarwal, Cho, Esteves, 2017). A highly condensed chromatin arranged in a toroid is formed when cysteine residues of protamines undergo intra‐ and intermolecular disulfide cross‐linking (Esteves, Agarwal, Majzoub, 2017; Ward & Coffey, 1991). This intricate packaging safeguards the sperm chromatin during transport through the male and female reproductive tracts and secures the transfer of intact paternal genome to the oocyte (Gawecka et al., 2015). In mammalian species, the quality of DNA packing relates to the number of cysteine residues at the protamine level; the higher the number of disulfide bonds, the higher the DNA stability (Gosálvez, López‐Fernández, et al., 2011). The DNA molecule would be subjected to a forced twisting if controlled DNA nicking—facilitated by topoisomerase II—had not taken place (Gosálvez, López‐Fernández, et al., 2015). Any process affecting protamination can disrupt chromatin condensation (Esteves, Agarwal, Cho, et al., 2017). Faulty chromatin compaction creates an abnormal tertiary chromatin structure that likely prevents the zygote from accessing the proper sequences of the paternal genome for the correct launch of the embryonic developmental programme (Dattilo et al., 2014). High levels of sperm nuclear chromatin condensation abnormalities have been related to decreased fertilisation rates, decreased embryo quality, elevated embryo development arrest and impaired pregnancy rates (Menezo et al., 2017). The most critical effect seems to be a block at the 2PN stage or even an absence of sperm nucleus' decondensation (Junca et al., 2012).

Failure to repair the DNA nicks—during histone to protamine replacement—can lead to persistent DNA breaks in viable ejaculated spermatozoa and/or trigger apoptosis. Moreover, defective chromatin maturation in the testis makes spermatozoa more susceptible to ROS attack during transit in the male genital tract, leading to sperm DNA breaks (Muratori et al., 2015). Nonetheless, viable spermatozoa with abnormal chromatin compaction andnonfragmented DNA can be released in the ejaculate (Gosálvez, López‐Fernández, et al., 2015; McPherson & Longo, 1993).

### 3.2 Apoptosis

Apoptotic markers like caspases, Fas, Bcl‐X, p53 and annexin V are present in mature spermatozoa, supporting apoptosis in the generation of DNA fragmentation. Double‐strand DNA breaks, controlled by specific DNases, degrade the DNA molecule when caspase or annexin V is detected on the sperm surface (Gorczyca et al., 1993; Muratori et al., 2000, 2015; Sakkas et al. 2002; Paasch et al., 2004). Despite this, the association between apoptotic markers and DNA fragmentation is not unequivocal (Moustafa et al. 2004). Moreover, the apoptotic processes leading to SDF might be different to some degree from the classic apoptotic pathways in somatic cells (Moustafa et al. 2004).

### 3.3 Oxidative stress

Oxidative stress resulting from excessive ROS production during sperm transit through the seminiferous tubules and epididymis has been regarded as the leading underlying causative factor for SDF (Ollero et al., 2001; Sakkas & Alvarez, 2010). Human spermatozoa are susceptible to OS due to the abundant polyunsaturated fatty acid content in plasma membranes. Besides, the sperm cytoplasm has limited cytosolic content of antioxidant factors. Furthermore, spermatozoa possess reduced DNA damage detection and repair mechanisms (Champroux et al., 2016; Dada, 2017). ROS attack not only sperm membranes but also nuclear and mitochondrial DNA (Gosálvez, López‐Fernández, et al., 2015; Muratori et al., 2015; Sakkas & Alvarez, 2010). Based on the oxidative attack amplitude, ROS can also damage the sperm nucleus by modifying bases, creating abasic sites, chromatin protein cross‐linking and DNA strand breaks (Gosálvez, Fernández, et al., 2015; Gosálvez, López‐Fernández, et al., 2015). Excessive ROS generates oxidised base adducts (e.g. 8‐oxo‐7,8‐dihydro2‐deoxyguanosine [8OHdG]), which are cleaved out of the DNA by an enzyme named 8‐oxoguanine DNA glycosylase 1 (OGG1) DNA, thus creating an unstable abasic site more vulnerable to fragmentation (Aitken, 2016; Feng et al., 2003; Lopes et al., 1998).

Oxygen radicals and physicochemical factors also activate endogenous caspases and endonucleases, thus acting as intrinsic factors causing SDF. It has been shown that spermatozoa from several species, including humans, have an endogenous nuclease that directly participates in apoptosis (Sotolongo et al., 2005). Moreover, the presence of DNase activity at the seminal plasma can be an additional source of DNA cleavage (Cortés‐Gutiérrez et al., 2019; Sotolongo et al., 2003).

Environmental and occupational toxicants (e.g. phthalate exposure, air pollution, high temperature), lifestyle (e.g. obesity; smoking), infection, fever, radiotherapy, chemotherapy and ageing have been related to SDF (Evenson et al., 2000, 2020; Jurewicz & Hanke, 2011; Jurewicz et al., 2009; O'Flaherty et al., 2008; Rubes et al., 2007; Schmid et al., 2007; Wyrobek et al., 2006). However, extrinsic factors' potential adverse effect is not universal, suggesting that genetic predisposition gives some individuals the ability to metabolise toxic products with increased efficiency (Chengyong et al., 2012; Evenson & Wixon, 2005; Rubes et al., 2007).

## 4 WHAT ARE THE SPERM DNA FRAGMENTATION TESTS AND HOW TESTING SHOULD BE PERFORMED AND INTERPRETED?

Sperm DNA fragmentation tests were initially developed to detect DNA damage in the spermatozoa of nonhuman species (reviewed by Evenson, 2016, 2017, 2018). A remarkable association between SDF and fecundity was demonstrated in bull/cow, stallion, and boar studies (Ballachey et al., 1987, 1988; Didion et al., 2009; Evenson et al., 1994; Kenney et al., 1995). In 1980, Evenson's landmark publication introduced the concept of SDF as related to pregnancy outcomes in humans (Evenson et al., 1980). The SDF rates (measured by the sperm chromatin structure assay [SCSA]) were twice as higher in patients attending infertility clinics than men of known fertility. This study also included pregnancy outcomes for bulls, showing that the SDF rates were four times higher in animals of known low fertility than those of high fertility.

Over the last 40 years, knowledge concerning SDF's impact on human fertility has increased steadily (see Translational Andrology and Urology (Sperm DNA Fragmentation), 2017). The development and clinical application of SDF tests indubitably represent one of the best examples of translational medicine in andrology. The term ‘DNA fragmentation’ is broadly used to refer to any chromatin damage; however, not all injuries break the DNA into ‘fragments’. Besides SS‐DBs and DS‐DBs, chromatin damage includes defective nuclear protein and altered chromatin configuration.

**Figure 1:**
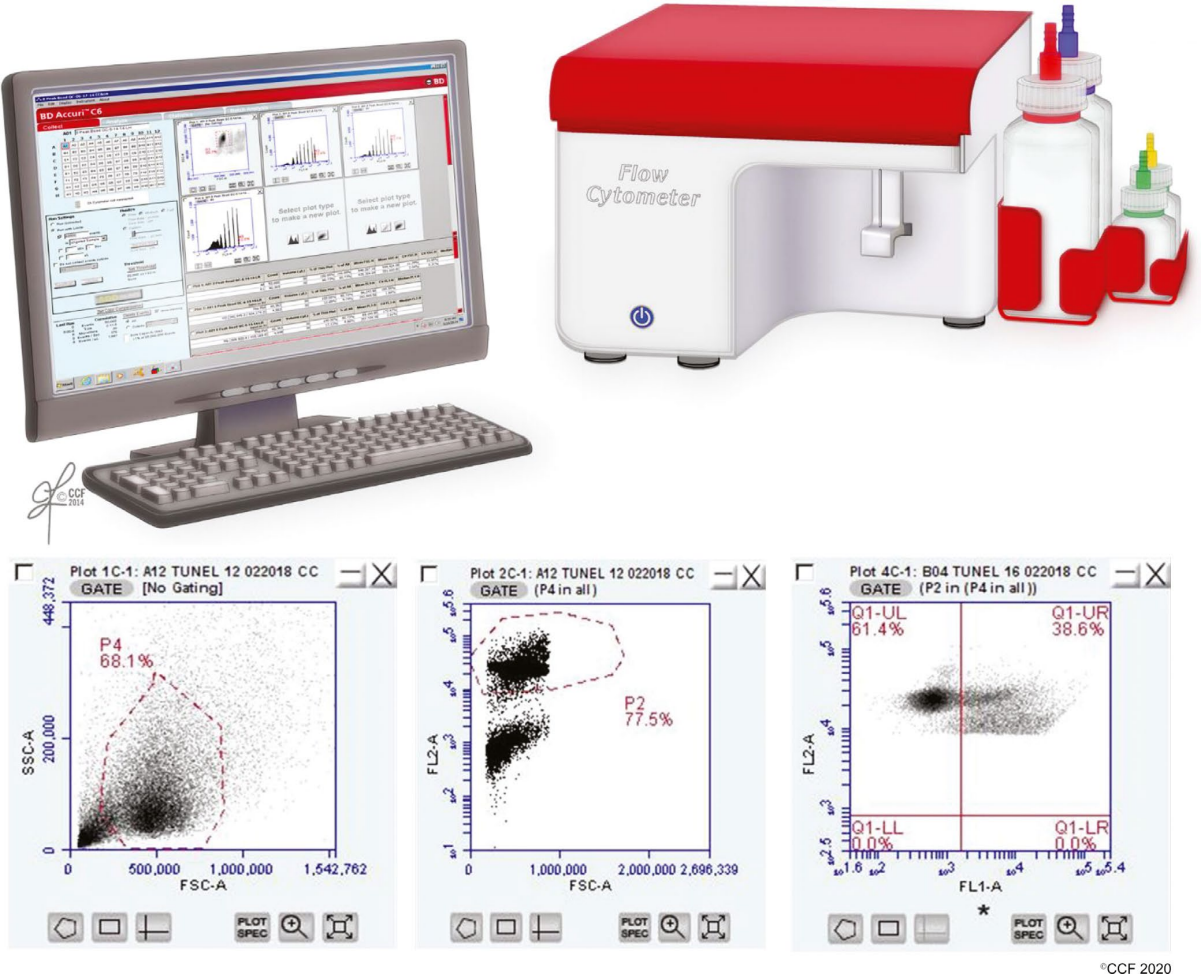
TUNEL assay (Flow Cytometry). TUNEL assay using BD Accuri C6 flow cytometer (top). Boxes (bottom panels) include representative plots of a positive sample. (a) Forward scatter versus side scatter or ‘Plot 1’: Gate is drawn, and small debris and larger nonsperm cells are excluded. Spermatozoa stained with propidium iodide (PI) with a flame‐shaped gate are gated in the forward scatter (FSC) versus side scatter (SSC) plot. (b) Gating strategy for PI positive cells. (c) Plot of a positive sample. SSSC‐A: Side scatter area; FSC‐A: forward scatter area; FL2‐A: fluorescence in the red or propidium iodide channel‐area; FL1‐A: fluorescence in the green or FITC‐area; Q1‐UR: Quadrant 1‐upper right; Q1‐UL: Quadrant 1‐ Upper Left; Q1‐LL: Quadrant 1‐Lower Left and Q1‐LR: Quadrant 1‐Lower Right. Asterisk indicates that virtual gain is applied to the data by aligning with the negative peak of a standard sample with known DNA fragmentation

**Figure 2:**
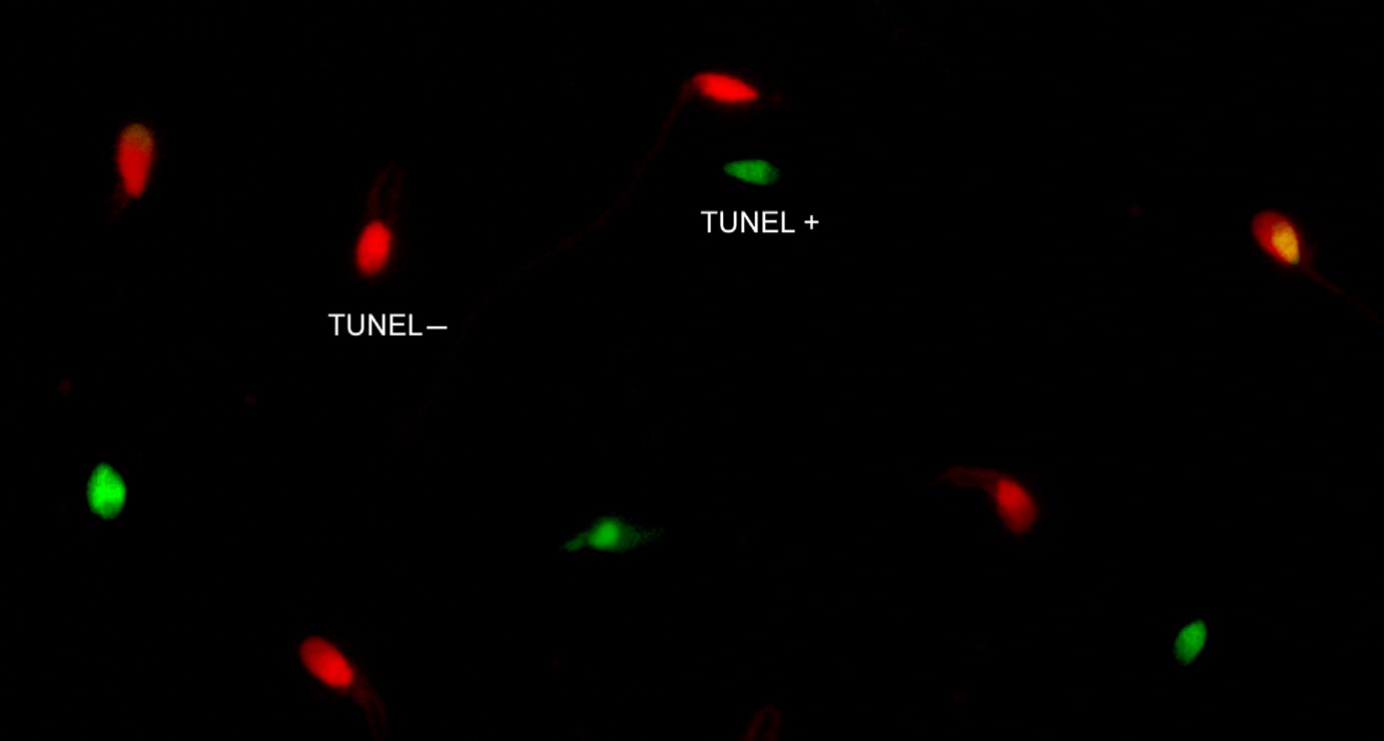
TUNEL Assay (Fluorescence Microscopy). Visualisation of sperm DNA damage using terminal deoxynucleotidyl transferase dUTP nick end labelling (TUNEL). Digoxigenin‐dUTP is incorporated to DNA breaks using a terminal transferase; anti‐digoxigenin‐FITC is used to label the sites where digoxigenin‐dUTP is present (green colour). TUNEL + represents spermatozoa presenting DNA damage. Slides were counterstained with propidium iodide (red colour). TUNEL‐ represents spermatozoa free of DNA breaks

**Figure 3:**
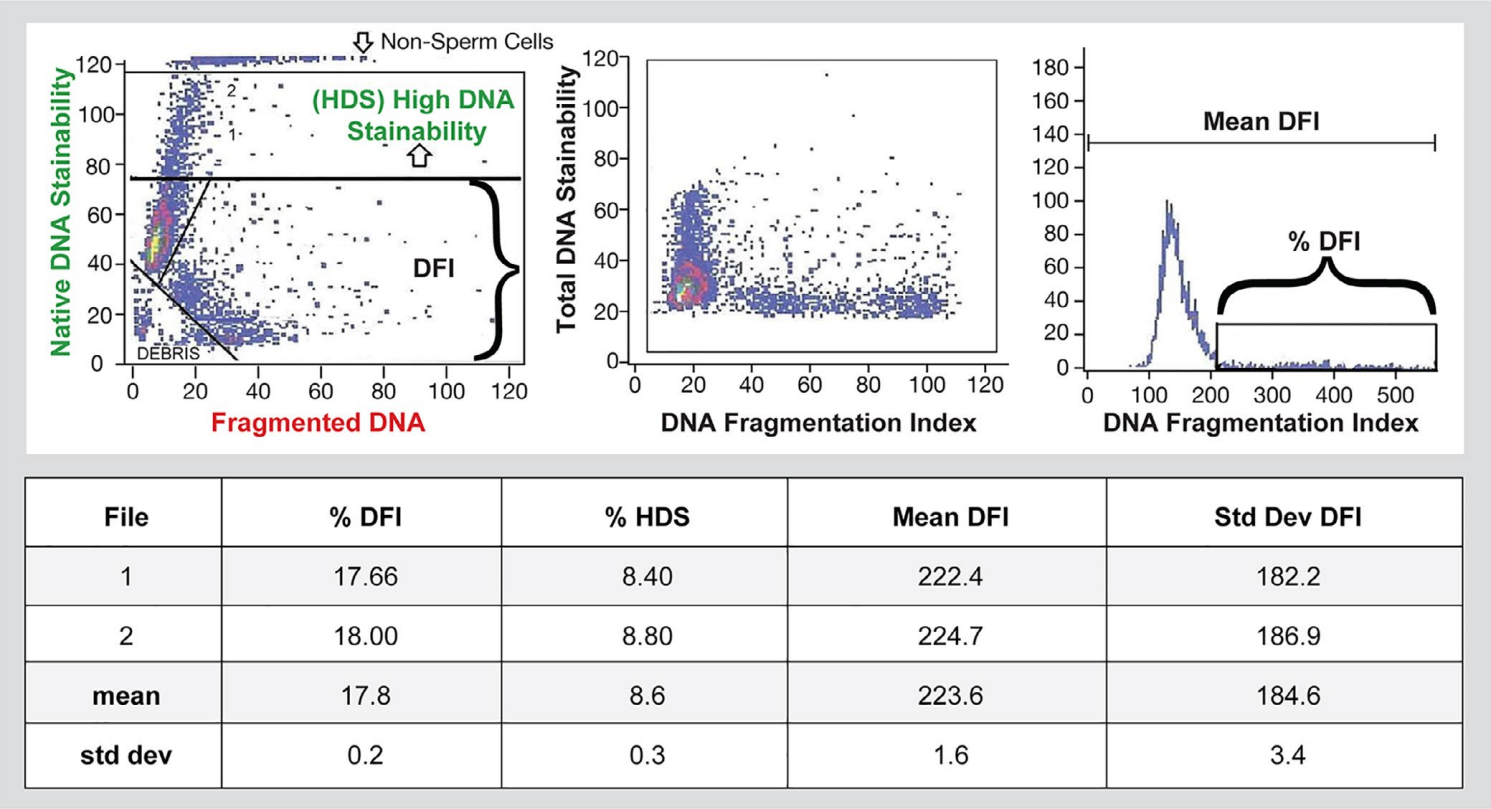
Sperm chromatin structure assay (SCSA). Test data (SCSA Diagnostics, Brookings, USA). Left panel (top box): raw data from a flow cytometer showing each of 5,000 spermatozoa as a single dot on a scattergram. *Y*‐axis = green fluorescence with 1,024 gradations (channels) of DNA stainability (intact double‐stranded DNA). *X*‐axis = red fluorescence with 1,024 gradations of red fluorescence (single‐strand DNA). Axes shown are 1,024/10. Line at Y = 75 marks the upper boundary of DNA staining of normal sperm chromatin; above that line are spermatozoa (dots) with partially uncondensed chromatin allowing more DNA stainability. Bottom left corner shows gating out of seminal debris. Middle panel: Raw data from left panel are converted by SCSAsoft software (or equivalent) to red/red + green fluorescence. This transforms the angled sperm display in the left panel to a vertical pattern that is often critical for accurately delineating the percentage of spermatozoa with fragmented DNA. *Y*‐axis = total DNA stainability versus. *X*‐axis = red/red + green fluorescence (DFI). Right panel: Frequency histogram of data from middle panel showing computer gating into %DFI and Mean DFI. Bottom box: SCSAsoft software calculations of mean of two independent measures of mean and standard deviation (std dev) of median DFI, %DFI and %HDS (high DNA stainability)

**Figure 4:**
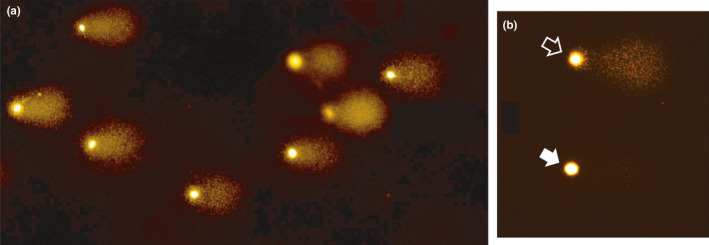
Alkaline Comet assay under fluorescence microscopy. (a) Sperm sample of a patient exhibiting elevated sperm DNA fragmentation (SDF). Several comets are shown which represent spermatozoa with DNA fragmentation. The longer and brighter the ‘Comet’ tail, the more fragmentation is present. (b) Spermatozoon with DNA fragmentation (open arrow), and another one with a hardly visible ‘Comet’ tail (white arrow), representing a cell with minimal DNA fragmentation. As the Comet test measures the amount of damage in each cell, it is rare to find a perfect spermatozoon with 0% damage, even from fertile donors

**Figure 5:**
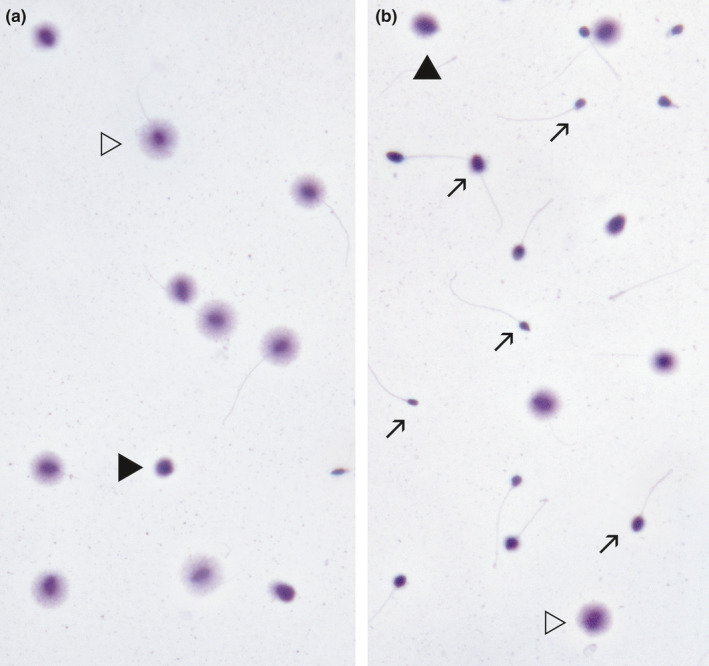
Sperm Chromatin Dispersion test (SCD) under bright‐field microscopy (Halosperm, Halotech DNA, SL, Madrid, Spain). (a) Sperm sample of an individual presenting with normal level of sperm DNA fragmentation (SDF). (b) Sperm sample of a patient with varicocele presenting with elevated SDF. Open arrowheads indicate spermatozoa with halos of dispersed chromatin representing a normal DNA molecule with no fragmented DNA. Black arrowheads indicate spermatozoa with small or absent halos of dispersed chromatin, representing spermatozoa with fragmented DNA. Arrows in ‘b’ indicate spermatozoa with fragmented‐degraded DNA

**Figure 6:**
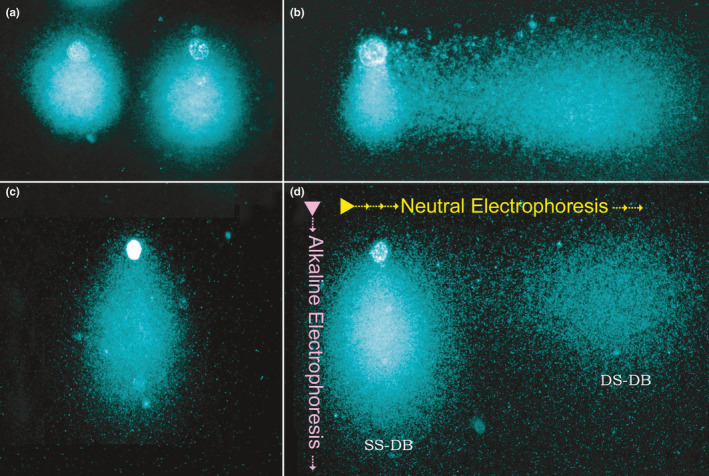
Two‐dimensional (two‐tail) Comet assay for simultaneous mapping of single‐strand DNA damage (SS‐DB; *Y*‐axis) and double‐strand DNA damage (DS‐DB; *X*‐axis) in spermatozoa. (a) Normal spermatozoa showing DNA displacement in the *Y*‐axis due to the structural presence of alkaline labile sites. (b) Presence of DS‐DB in the *X*‐axis after electrophoresis under neutral conditions. (c) Presence of SS‐DB along the *Y*‐axis after alkaline electrophoresis. (d) Presence of both SS‐DB and DS‐DB affecting a single spermatozoon. Arrows indicate the perpendicular sense of each electrophoresis

The existing tests can be group in methods that use (a) enzymatic reactions to label the DNA breaks, (b) controlled DNA denaturation combined with protein depletion as intermediates to reveal the DNA breaks and (c) dyes that bind to relaxed GC‐rich motifs. The first category comprises tests that utilise a terminal transferase (e.g. Terminal deoxynucleotidyl transferase‐mediated dUTP‐biotin nick end labelling; TUNEL; Figures 1 and 2) or specific enzymes such as the Klenow fragment (e.g. in situ nick translation assay; ISNT) to label the free 3–OH ends of the nucleotide at the DNA break. In the latter, a 5′→3′ polymerase activity is combined with a 3′→5′ exonuclease activity for the elimination of precoding nucleotides and proofreading. Both TUNEL and ISNT assays detect single‐stranded (SS‐DB) and double‐stranded DNA breaks (DS‐DB) indistinctively. The second category includes tests that apply DNA denaturation and/or controlled protein depletion. Within this group, the SCSA relies on controlled DNA denaturation to target pre‐existing DNA breaks (Figure 3). The alkaline Comet assay (Figure 4) and the sperm chromatin dispersion test (SCD; Figure 5) are based on DNA denaturation and controlled protein depletion. Like TUNEL, these assays determine the global SDF without discriminating between spermatozoa with SS‐DBs or DS‐DBs. By contrast, the neutral Comet assay only uses a controlled protein depletion to exclusively detect DS‐DB whereas the 2‐dimensional Comet assay applies two electrophoretic runs—one in a neutral buffer and another in an alkaline buffer—to map SS‐DBs and DS‐DBs simultaneously (Figure 6; Cortés‐Gutiérrez et al., 2017). The third category includes tests that detect abnormal chromatin packaging using the fluorescent antibiotic chromomycin A3 staining, given its preference to bind relaxed DNA GC‐rich motifs, toluidine blue staining, acridine orange test and aniline blue staining (reviewed by Gosálvez, López‐Fernández, et al., 2015). Notably, given that histone‐complexed DNA —stained by acridine orange—fluoresces twice as likely as protamine‐complexed DNA (Evenson et al., 1986), this high DNA stainability sperm fraction, which represents spermatozoa with excess nuclear histones and faulty chromatin condensation, can also be detected by the SCSA. Hence, this test also provides information about chromatin compaction (HDS; see Figure S1).

Thus, the information provided by each assay does not necessarily line up. Abnormal nucleus condensation is primarily associated with protamine deficiency or protamine mispackage via broken DNA‐protein ionic links; by contrast, DNA fragmentation primarily relates to oxidative stress (Aitken, 2017a; Gosálvez, López‐Fernández, et al., 2015; Menezo et al., 2017; Muratori et al., 2015; Ribas‐Maynou & Benet, 2019).

On this basis, we propose a new nomenclature to embrace the tests into two groups, that is, (1) SDF tests, namely TUNEL, ISNT, SCSA, SCD, and Comet, and (2) Sperm chromatin compaction tests, namely, chromomycin A3 staining, acridine orange staining, toluidine blue staining and aniline blue staining.

Tests that measure SDF (e.g. TUNEL, SCSA, SCD and Comet) may be preferable to those that measure chromatin compaction due to the role of oxidative stress in male infertility (Esteves, Agarwal, Majzoub, 2017; Gosálvez, López‐Fernández, et al., 2015). However, assessment of chromatin compaction has been suggested to be clinically useful to patients with unexplained infertility and RPL if the results of an SDF test are unremarkable (Evenson, 2016; Evenson et al., 2020).

Table 1 summarises the SDF tests′ characteristics, namely, TUNEL, SCSA, SCD and alkaline Comet. These are the most commonly requested tests by practitioners (Majzoub, Agarwal, Cho, Esteves, 2017). Each test may have different clinical thresholds due to the different DNA damage sites detected and the particular technical aspects of each assay (Gawecka et al., 2015). An in‐depth analysis of standardisation, cut‐off values, reproducibility and limitations of existing tests is beyond this paper; this information can be found elsewhere (Esteves, Agarwal, Cho, et al., 2017; Esteves, et al., 2020; Ribas‐Maynou et al., 2013; Santi et al., 2018).

**Table 1:** Characteristics of sperm DNA fragmentation assays

Test	Acronym	Principle	Specimen requirements	Thresholds^a^			Can be done in surgically retrieved specimens (e.g. testicular spermatozoa)?
				Male infertility diagnosis	IUI (likelihood of pregnancy and/or miscarriage)	IVF/ICSI (likelihood of pregnancy and/or miscarriage)	
Terminal deoxynucleotidyl transferase‐mediated dUTP‐biotin nick end labelling	TUNEL	The assay relies on a terminal deoxynucleotidyl transferase (TdTA) enzyme for the ‘direct’ labelling of 3′ free ends of DNA. The sites of breaks can be identified with the aid of optical fluorescence microscopy or flow cytometry. The TUNEL test essentially assays the protamine toroid linker DNA as the relatively large TdTA molecule might not penetrate the highly compacted protamine toroid. Protocol modifications have been introduced to increase its sensitivity, including DNA decompaction using dithiothreitol (DTT)	Whole ejaculate, 2.0–2.5 × 10^6^ spermatozoa for flow cytometry. 10,000 cells/events are counted. With microscopy, a total of 500 spermatozoa are assessed.	>17% by flow cytometry^1^	No thresholds defined for neat semen; >12% for post‐wash specimen using fluorescence microscopy^2^	No clear thresholds using flow cytometry established. >36% using fluorescence microscopy^3,4^	Yes, using fluorescence microscopy only.^5^
Sperm Chromatin Structure Assay	SCSA	The assay is based on acid denaturation of the DNA at the sites of existing single‐ or double‐strand breaks. Acridine orange (AO) is used for staining; the dye penetrates the sperm chromatin and intercalates into double‐stranded DNA (intact DNA), which fluoresces green when exposed to blue laser light. In contrast, AO attachment to single‐strand DNA creates a complex that produces a metachromatic shift to red fluorescence. The red fluorescence represents DNA strands that originate from the sites of single‐ or double‐strand breaks. The fluorescence patterns emitted by spermatozoa are captured using a flow cytometer, and the ratio of red to total (green + red) fluorescence intensity is used to calculate the percentage of spermatozoa with DNA fragmentation (DFI; DNA fragmentation index).	>0.5 million sperm/ml raw semen for direct measurement. If <0.5 million/ml, specimens should be concentrated by centrifugation and resuspended. Specimens are shipped to the reference laboratory as ~0.2 ml frozen raw semen aliquot in boxed dry ice or LN2 dry shipper.	>20%–25%^6^ (DFI) >20%–25% (HDS)	>25%^7–9^ (DFI) >25% (HDS)	>30%–35%^7–9^ (DFI) >25% (HDS)	Yes, if sufficient number of spermatozoa.
Sperm Chromatin Dispersion	SCD	The assay relies on the principle that spermatozoa with DNA fragmentation fail to produce the characteristic halo of dispersed DNA loops that are observed in spermatozoa with nonfragmented DNA, following acid denaturation and removal of nuclear proteins. Sperm suspensions are embedded in agarose gel on slides and treated with an acid denaturation solution (HCl) to generate restricted single‐strand DNA motifs at the sites of existing single‐ or double‐strand breaks. The denaturation is stopped, and spermatozoa are exposed to a lysing solution based on DTT, sodium dodecyl sulphate, and NaCl to remove the sperm membrane and nuclear proteins. Then, slides are stained with DAPI (4′,6‐diamidino‐2‐phenylindole) or Diff‐Quik, and spermatozoa with nondispersed and dispersed chromatin loops are identified by fluorescence or bright‐field microscopy examination,respectively, to calculate the percentage of sperm with DNA fragmentation. The halos correspond to relaxed DNA loops attached to the residual nuclear structure as seen in spermatozoa with low or no SDF. By contrast, spermatozoa with very small or no halos correspond to those exhibiting SDF as confirmed by DNA breakage detection‐fluorescence in situ hybridisation, a procedure in which the restricted single‐stranded DNA motifs generated from DNA breaks can be detected and quantified.	The test can be performed in a single to several hundred spermatozoa, both in fresh and frozen‐thawed specimens. The recommended sperm concentration is 1–3 million per ml, but it can be performed using less concentrated samples.	In general, thresholds ranging from >16% to 30% are used for male infertility diagnosis, with 20% considered the value that best discriminates infertile from fertile men.^6^ Using sperm donors and patients attending an infertility clinic, a threshold of >16% was found to best discriminate both cohorts with an AUC of 0.874, specificity of 85% and specificity of 75%.^10^	20%^11^	Using oocyte donor cycles to control for female factors, a 25% threshold discriminates between clinical pregnancy and no pregnancy, with an AUC of 0.711.^12^	Yes. The SCD test can be used on PESA, MESA, TESA or TESE obtained spermatozoa as well as low concentrated samples derived from IMSI, PICSI, MACS or microfluidics. In TESA/TESE specimens, somatic cells and spermatozoa can be distinguished using a dual combination of fluorochromes with DNA and protein specificity.^13^
Single‐cell gel electrophoresis assay	Comet	The assay relies on DNA decompaction and protein depletion coupled to single‐cell electrophoresis in agarose microgel. Removal of protamines and histones creates a nucleoid‐like structure containing supercoiled loops of DNA. Alkaline or neutral pH conditions allow the uncoil of double‐stranded DNA, which under electrophoresis results in migration of fragments of single‐ and double‐stranded DNA towards the anode, thus forming a comet tail that can be observed under fluorescence microscopy. The relative fluorescence in the tail compared with its head reflects the level of SDF; spermatozoa with increased fluorescence intensity in the comet tails have high levels of chromatin damage. Additional quantitative parameters can be used to increase the test′s precision, such as nucleus diameter, olive tail movement and comet length. The alkaline Comet assay detects global SDF, i.e., both single‐ and double‐stranded DNA breaks, as well as alkali‐labile sites, whereas the neutral Comet assay detects double‐stranded DNA breaks only. Alternatively, the two‐tailed Comet assay can be used to assess and differentiate the type of break in the same spermatozoon. The assay firstly applies neutral lysis and electrophoresis to detect double‐strand breaks, and then, by turning the slide 90º and applying alkaline lysis and electrophoresis, single‐strand breaks are detected. The Comet assay provides three parameters: ACS: average SDF across 100 individual spermatozoa; HCS: the proportion of spermatozoa with high DNA damage; LCS: the proportion of spermatozoa with low DNA damage.	A minimum of 100 µl volume of neat semen containing a minimum of 5,000 spermatozoa.	>26% (ACS)^14,15^ <74% (LCS) >4% (HCS) AUC of these parameters = 0.909–0.936^14,15^	>26% (ACS)^15^ <70% (LCS) >2% (HCS) AUC of these parameters = 0.883–0.969^15^	>27% (ACS)^14,15^ <68% >10%	Yes; the Comet assay can be used on PESA, MESA, TESA or TESE obtained spermatozoa as well as low concentrated samples derived from IMSI, PICSI, MACS or microfluidics.

Note

^1^Sharma, Ahmad, et al. (2016); ^2^Duran et al. (2002); ^3^Henkel et al. (2004); ^4^Frydman et al. (2008); ^5^Xie et al. (2020); ^6^Santi et al. (2018); ^7^Bungum et al. (2004); ^8^Bungum et al. (2007); ^9^Evenson (2013); ^10^Gosálvez, et al. (2015); ^11^Vandekerckhove et al. (2016); ^12^Gosálvez et al. (2013); ^13^Esteves, et al. (2015); ^14^Nicopoullos et al. (2019) (see reference list); ^15^Universally recognised as an adequate number of Comets in any cell type to accurately reflect the damage across the entire population; Clinical thresholds for male infertility are based on comparisons with spermatozoa from 1,000 fertile donors; Clinical thresholds for miscarriage are based on comparisons of 100 fertile donors and 217 couples experiencing miscarriage; Clinical thresholds for IVF/ICSI are based on 381 ART cycles (77 IVF and 226 ICSI), with comparisons between those couples who achieved a live birth and those who did not.

Abbreviations: DTT: dithiothreitol; ACS: average Comet score; LCS: low Comet score; HCS: high Comet score; SDF: sperm DNA fragmentation; DFI: DNA fragmentation index; HCS: high chromatin stainability; AUC: area under the curve; IUI: intrauterine insemination; IVF: in vitro fertilisation; ICSI: intracytoplasmic sperm injection; PESA: percutaneous epididymal sperm aspiration; MESA: microsurgical epididymal sperm aspiration; TESA: testicular sperm aspiration; TESE: testicular sperm extraction; PICSI: physiological hyaluronan selected spermatozoa for ICSI; IMSI: high‐magnification selected spermatozoa for ICSI; MACS: sperm selection using magnetic‐activated cell sorting.

^a^ Neat semen.

Briefly, SDF measured in consecutive ejaculates seems to have low biological variability (Evenson et al., 1991; Zini et al., 2001). In one study evaluating SDF rates (by SCSA) in consecutive ejaculates, the variation was remarkably lower (~9%) than conventional semen parameters (range: 28%–43% in count, motility, and morphology). Moreover, inter‐ and intra‐observer coefficients of variation, computed for SCSA, SCD and TUNEL (using flow cytometry), are reported to be below 10% (Fernández et al., 2005; Giwercman et al., 2003; McEvoy et al., 2014; Sharma et al., 2010; Sharma, Ahmad, et al., 2016). Interlaboratory agreement is very high (*r* > 0.9) for SDF measured using the SCSA (Evenson et al., 1995; Evenson, 2018) or the flow cytometry TUNEL assay (Ribeiro et al., 2017). Lastly, although results provided by the four most common tests are not necessarily aligned, there seems to be a good correlation among them (Javed et al., 2019; Ribas‐Maynou et al., 2013). In one study, high correlations were found between SCD and SCSA (*r* = 0.71; *p* < 0.001), SCD and TUNEL (*r* = 0.70; *p* < 0.001), and SCSA and TUNEL (*r* = 0.79; *p* < 0.001), whereas moderate correlations were reported for alkaline Comet and SCD (*r* = 0.61; *p* < 0.001), alkaline Comet and SCSA (*r* = 0.59; *p* < 0.001), and alkaline Comet and TUNEL (*r* = 0.72; *p* < 0.001; Ribas‐Maynou et al., 2013).

Testing should be carried out in the neat semen after an ejaculatory period of 2–5 days. SDF results may increase significantly as a function of abstinence length (Agarwal, Gupta, et al., 2016; Gosálvez, González‐Martínez, et al., 2011; Hanson et al., 2018); therefore, a fixed abstinence period should be used, in particular, to monitor the results of medical or surgical interventions aimed at decreasing SDF (Esteves, Santi, et al., 2020). Moreover, it is suggested that patients have 1–2 ejaculations during the week before the test. This advice relates to the fact that the epididymis does not empty all spermatozoa with a single ejaculation (Misell et al., 2006); thus, if a patient had not had an ejaculation for an extended period, it is likely that some dead and apoptotic spermatozoa would be released with the new ejaculation.

The time elapsed between ejaculation and testing is critical as SDF rates can increase as a function of time post‐ejaculation in an individual‐dependent manner (Gosálvez et al., 2009). In experiments using semen donors, SDF rates (assessed by the SCD test) increased remarkably during the first post‐ejaculation hours in the neat semen, and also in frozen‐thawed specimens incubated with culture medium (Tvrdá et al., 2018). On this basis, it is suggested that analysis is started as quickly as possible after liquefaction (e.g. 30–60 min in neat semen) or immediately after thawing if the test requires freezing for later SDF assessment. In the latter, immediate specimen freezing should be done after liquefaction is achieved.

Experiments on rodents and humans have shown that SDF data measured by SCSA, TUNEL, SCD and Comet in extender are similar to those obtained from specimens that were flash‐frozen in liquid nitrogen (Evenson et al., 1994; McEvoy et al., 2014; Young et al., 2003). However, the evidence is not unequivocal, as some studies show that the type of cryomedia, cryopreservation technique and semen quality might influence post‐thaw SDF rates (versus baseline values; Kopeika et al., 2015; Lusignan et al., 2018; Raad et al., 2018; reviewed by Paoli et al., 2019).

The clinical utility of assessing SDF in processed semen (e.g. after gradient centrifugation or swim‐up) is not supported by current evidence, as results cannot predict the likelihood of pregnancy (Bungum et al., 2008; Niu et al., 2011). Moreover, gradient centrifugation might increase SDF in some cases, thus adversely impacting ART pregnancy outcomes, potentially (Muratori et al., 2016; Zini et al., 2000).

Although the best assay to quantify SDF and its optimal thresholds are still to be defined, the four major SDF tests mentioned above (SCSA, Comet, SCD, and TUNEL) provide reliable information about sperm DNA integrity in subfertility. However, it is vital to understand how each test reports results.

In SCD and conventional TUNEL, the assessments are carried out manually on one to several hundred spermatozoa, under bright‐field (SCD) or fluorescence microscopy (SCD; TUNEL; see Figures 2 and 5), and the number of spermatozoa exhibiting DNA fragmentation—relative to the total number of spermatozoa analysed—represents the %SDF (Feijó & Esteves, 2014; Fernández et al., 2005).

The flow cytometry TUNEL and SCSA measure the extent of SDF across 5,000–10,000 spermatozoa. These assays report the percent of cells with broken DNA (dots on scatter plot; see Figures 1 and 3) and provide data on the amount of SDF in every cell. For instance, the SCSA, in addition to the %DFI (DNA fragmentation index), reports the mean DFI (Figure 3); this measure represents the entire amount of DNA fragmentation measured by flow cytometer channels. Since the %DFI and mean DFI are highly correlated (Evenson et al., 2020), this indicates that the commonly used %DFI is also a measure of the total DNA fragmentation in a given semen sample (see Figures S1–S5, for examples of tests′ reports).

The Comet assay measures DNA fragmentation in each cell using a semi‐automated or automated system (Albert et al., 2016; Nicopoullos et al., 2019). In Comet, the average Comet score (ACS) represents the average amount of DNA fragmentation across 100 individual comets (spermatozoa) analysed; the proportion of comets with low and high DNA fragmentation is also reported to provide additional discriminatory information (see Figure S2). Accordingly, although SCD and alkaline Comet have similar clinical thresholds (25%–27%) for IVF/ICSI, a 27% value by SCD means that 27% of the spermatozoa analysed have DNA fragmentation, whereas 73% had no detectable damage. By contrast, the same value by alkaline Comet indicates that the average amount of damage per spermatozoon was 27% in the analysed specimen.

A systematic review and meta‐analysis of 28 studies indicated that thresholds of 20% (considering mainly SCSA, TUNEL and SCD) best discriminate confirmed and presumed fertile men from infertile men, with an area under the curve (AUC) of 0.844 (sensitivity: 79%; specificity: 86%; Santi et al., 2018). This threshold also held when only TUNEL studies were combined (15 studies; AUC: 0.831, *p* = 0.002). However, thresholds may vary slightly according to individual studies and methods. For instance, a clinical threshold of 17% has been reported for male infertility diagnosis with the flow cytometry TUNEL assay (Sharma, Ahmad, et al., 2016) and 16% for SCD (Gosálvez, Fernández, et al., 2015).

Along these lines, clinical thresholds of 26 % by alkaline Comet (average Comet score) discriminate fertile from infertile men with an AUC of 0.925 (sensitivity: 73%; specificity: 100%; Nicopoullos et al., 2019).

Moreover, SDF values greater than 20%–30% (by SCSA, alkaline Comet and SCD, obtained in neat semen) are clinically useful for classifying infertile couples into a statistical probability of prolonged time to achieve natural pregnancy, decreased likelihood of pregnancy by IUI, IVF or ICSI and increased risk of miscarriage (Bungum et al., 2008; Evenson, 2013; Gosálvez et al., 2013; Majzoub, Agarwal, Cho, Esteves 2017; Nicopoullos et al., 2019; Oleszczuk et al., 2016; Vandekerckhove et al., 2016). These clinical thresholds seem to hold for ICSI cycles using donor oocytes (Gosálvez et al., 2013).

In contrast, TUNEL clinical thresholds for IUI, IVF and ICSI have yielded mixed results, with values ranging from 10% to 36% (Benchaib et al., 2007; Borini et al., 2006; Cho et al., 2017a; Duran et al., 2002; Frydman et al., 2008). However, the TUNEL studies are not homogenous concerning the SDF measurements, as most of them utilised post‐thaw SDF values, which are not predictive of IVF/ICSI outcomes, as previously mentioned. When only studies utilising neat semen are examined, a TUNEL clinical threshold of ~36% seems optimal to determine the reproductive success probability among couples undergoing IVF/ICSI (Frydman et al., 2008; Henkel et al., 2004).

Table 2 summarises the evidence concerning SDF testing methods, clinical thresholds and test results′ interpretation. Overall, test results obtained by assessing the neat semen provide information about sperm quality as a whole, not only the damaged spermatozoa unmasked by the assay.

**Table 2:** Sperm DNA fragmentation testing: methods, thresholds and interpretation

1. Methods	
In the male evaluation, given the ubiquity of oxidative stress contributing to male infertility, tests that measure SDF (e.g. TUNEL, SCSA, SCD and Comet) may be preferred over those that assess chromatin compaction because the former are more specific to detect oxidatively induced DNA damage.	Muratori et al. (2015) Gosálvez, et al. (2015) Aitken (2017) Menezo et al. (2017) Ribas‐Maynou and Benet (2019) Esteves, Santi, et al. (2020)
SDF measured in consecutive ejaculates has low biological variability.	Evenson et al. (1991) Zini et al. (2001) Smit et al. (2007)
Intra‐ and interlaboratory agreement is high for SDF measurements performed with SCSA, SCD and flow‐cytometer TUNEL.	Evenson et al. (1995) Giwercman et al. (2003) Fernández et al. (2005) Sharma et al. (2010) McEvoy et al. (2014) Sharma, Ahmad, et al. (2016) Evenson (2018)
Although the results provided by the most common SDF tests do not necessarily line up, there is a good correlation between SDF rates reported by TUNEL, SCSA, SCD and alkaline Comet.	Ribas‐Maynou et al. (2013) Javed et al. (2019)
SDF increases as a function of abstinence length.	Gosálvez, et al. (2011) Agarwal, et al. (2016) Hanson et al. (2018)
The time elapsed between ejaculation and testing, and specimen′s thawing and testing may affect SDF rates. SDF rates can increase as a function of time post‐ejaculation in an individual‐dependent manner.	Gosálvez et al. (2009) Tvrdá et al. (2018)
Animal and human studies indicate that SDF can be assessed in frozen‐thawed specimens as results obtained from fresh or flash‐frozen specimens by SCSA, TUNEL, SCD and alkaline Comet tend to be similar. However, some studies demonstrate that post‐thaw SDF rates might be increased (versus baseline values) depending on the type of cryomedia, cryopreservation technique and semen quality.	Evenson et al. (1994) Young et al. (2003) McEvoy et al. (2014) Kopeika et al. (2015) Lusignan et al. (2018) Raad et al. (2018) Paoli et al. (2019)
2. Thresholds and Interpretation	
In SCSA, TUNEL and SCD, the number of spermatozoa with DNA fragmentation—relative to the total number of spermatozoa analysed—indicates the SDF rate (termed DFI in SCSA). The Comet assay quantifies the amount of DNA fragmentation in each cell. In Comet, the average Comet score (ACS) represents the average amount of DNA fragmentation across 100 individual cells analysed.	Fernández et al. (2005) Sharma, Ahmad, et al. (2016) Evenson et al. (2020) Nicopoullos et al. (2019)
Overall, SDF test results provide information about sperm quality as a whole. However, each assay may have different clinical thresholds owing to the different sites of DNA damage detected and the inherent technical aspects of each assay.	Gawecka et al. (2015) Gosálvez, et al. (2015) Esteves, Agarwal, Majzoub (2017) Evenson (2018)
SDF tests cannot perfectly discriminate fertile from infertile men.	Sharma et al. (2010) Sakkas and Alvarez (2010) Gosálvez, et al. (2015) Santi et al. (2018) Nicopoullos et al. (2019)
SDF tests cannot perfectly discriminate couples that will have a successful IUI, IVF or ICSI cycle from those that will not.	Duran et al. (2002) Henkel et al. (2004) Borini et al. (2006) Benchaib et al. (2007) Bungum et al. (2008) Frydman et al. (2008) Evenson (2013) Gosálvez et al. (2013) Vandekerckhove et al. (2016) Cho et al. (2017a) Majzoub, Agarwal, Cho, Esteves, (2017) Simon et al. (2017) Nicopoullos et al. (2019)
The predictive power of SDF tests is influenced by type (SS‐DB or DS‐DB), site (intron or exons) and extent of damage in each cell, as well as the number of affected cells and oocyte′s ability to repair SDF after fertilisation.	Zini and Sigman (2009) Sakkas and Alvarez (2010) Jin et al. (2015) Gosálvez, et al. (2015) Liang et al. (2019) Esteves (2020)
Thresholds of about 20% evaluated by TUNEL, SCSA, SCD and alkaline Comet, assessed on neat semen, best discriminate fertile from infertile men.	Santi et al. (2018)
Thresholds of 20%–30% evaluated by SCSA, alkaline Comet and SCD, assessed on neat semen, are clinically useful for classifying infertile couples into a statistical probability of longer time to achieve natural pregnancy, decreased chances of pregnancy by IUI, IVF and ICSI, and increased miscarriage risk.	Bungum et al. (2008) Evenson (2013) Gosálvez et al. (2013) Vandekerckhove et al. (2016) Majzoub, Agarwal, Cho, Esteves (2017) Simon et al. (2017) Nicopoullos et al. (2019)
Female age seems to modulate the effect of SDF on the probability of pregnancy in couples undergoing IVF/ICSI.	Jin et al. (2015) Liang et al. (2019)
SDF rates in processed semen (e.g. after gradient centrifugation or swim‐up) have low predictive power for the likelihood of successful pregnancy.	Zini et al. (2000) Bungum et al. (2008) Niu et al. (2011) Muratori et al. (2016)

This implies that the remaining spermatozoa in a given specimen, that is, those without detectable DNA fragmentation, are not necessarily free of damage. The ‘iceberg effect’ hypothesis was initially proposed by Evenson (Evenson et al., 2002) and Alvarez (Alvarez, 2005) and elaborated further by Gosálvez (Gosálvez et al., 2013). According to this hypothesis, SDF tests can detect spermatozoa with evident DNA fragmentation, but spermatozoa with undetectable damage may remain hidden or cryptic within that population. The latter might not have yet fully expressed SDF at the time of analysis, representing spermatozoa with a DNA fragmentation predisposition. The oxidative attack on sperm DNA can lead to the formation of oxidised bases, making the DNA strand prone to fragmentation. These spermatozoa are essentially cryptic in terms of SDF detection, waiting ‘under the surface', ultimately to be detected, depending on the degree of damage imposed, for instance, by ex‐vivo manipulation or iatrogenic damage before use in ART.

Indeed, it has been shown that the decrease in SDF rates seen after the use of sperm selection techniques for ART does not necessarily translate to improvements in pregnancy rates (De Geyter et al., 2019; Gosálvez et al., 2013). Moreover, a few double‐strand DNA breaks are sufficient to delay cell cycle progression (van den Berg et al., 2018). It is, therefore, suggested that this cryptic subpopulation may contain undetectable DNA damage (using current methods) that are lethal enough to impact reproductive success. Accordingly, the dynamic assessment of SDF by incubating spermatozoa in vitro and assessing SDF at different time points has been proposed as a way to detect the above mentioned sperm population (Tvrdá et al., 2018).

As with conventional semen analysis, SDF tests cannot perfectly discriminate fertile from infertile men or couples that will have a successful ART cycle from those that will not. Both partners can contribute to a couple′s infertility; thus, any test′s usefulness is also dependent on the other partner′s fertility. Before testing, clinicians should understand the characteristics of SDF assays (e.g. sensitivity and specificity, positive and negative predictive value; Zini & Sigman, 2009). The predictive power of SDF tests is influenced by type (SS‐DB or DS‐DB), site (intron or exons) and amount of damage in each cell, as well as the number of affected cells and the oocyte′s ability to repair SDF after fertilisation (Esteves, 2020; Jin et al., 2015; Liang et al., 2019; Sakkas & Alvarez, 2010). It seems plausible that different assays might be complementing each other in different clinical settings. Hence, clinical decisions must take into account the technical shortcomings of the assays.

## 5 WHEN IS SPERM DNA FRAGMENTATION TESTING WARRANTED?

Given the critical role of sperm DNA integrity for normal fertilisation, healthy embryo development and successful reproductive outcomes, SDF assessment has been used to acquire information about sperm quality at the molecular level (Esteves et al., 2011). This section summarises the best available evidence concerning the impact of SDF in usual clinical infertility scenarios (Table 3). Furthermore, we critically appraise the situations in which SDF testing could help identify the origin of the infertility condition and possibly guide therapeutic strategies.

**Table 3:** Sperm DNA fragmentation testing: Indications, rationale and evidence

3. Varicocele	
There is a significant association between clinical varicocele and SDF; approximately 50% of individuals with clinical varicocele have abnormal SDF levels.	Werthman et al. (2008) Moskovtsev et al. (2009) Zini and Dohle (2011) Agarwal et al. (2012) Esteves et al. (2012) Esteves, Gosálvez et al. 2015 Roque and Esteves (2018)
Varicocele repair decreases SDF rates.	Hamada et al. (2013) Roque and Esteves (2018)
Reduction in SDF rates after varicocele repair may translate in improved pregnancy rates.	Smit, Romijn, et al. (2010) Ni et al. (2014) Mohammed et al. (2015)
Reduction in SDF rates in all grades of clinical varicocele has been reported after varicocele repair, particularly grades 2 and 3.	Sadek et al. (2011) Krishna Reddy et al. (2015) Ni et al. (2014) and Ni et al. (2016) Abdelbaki et al. (2017) Zaazaa et al. (2018)
Varicocele repair does not seem to improve SDF rates in men with subclinical varicocele.	García‐Peiró et al. (2014) Ni et al. (2016)
4. Unexplained and Idiopathic Infertility	
Abnormal SDF levels are found in up to 20% of men with unexplained infertility (i.e. infertility despite no identifiable causative factor and normal routine semen parameters).	Saleh et al. (2003) Oleszczuk et al. (2013) Feijó and Esteves (2014) Gosálvez, et al. (2015) Santi et al. (2018) Gill et al. (2019) Esteves, Santi, et al. (2020)
Abnormal SDF levels are found in up to 40%–50% of men with idiopathic infertility (i.e. abnormal routine semen analysis and no identified causative factor).	Simon et al. (2013) Aktan et al. (2013) Le et al. (2019) Homa et al. (2019) Gill et al. (2019)
SDF is an independent predictor of male fertility status and chances of achieving natural pregnancy.	Evenson et al. (1999) Evenson and Wixon (2008) Oleszczuk et al. (2013) Buck Louis et al. (2014) Malić Vončina et al. (2016)
5. Recurrent Pregnancy Loss	
Abnormal SDF levels increases the likelihood of recurrent pregnancy loss (i.e. two or more pregnancy losses) after natural and assisted conception.	Zidi‐Jrah et al. (2016) Carlini et al. (2017) McQueen et al. (2019) Tan et al. (2019)
6. Intrauterine Insemination	
Abnormal SDF levels negatively affect pregnancy rates by IUI.	Duran et al. (2002) Bungum et al. (2004), Bungum et al. (2007) Rilcheva et al. (2016) Vandekerckhove et al. (2016) Chen et al. (2019) Sugihara et al. (2020)
7. In Vitro Fertilisation/Intracytoplasmic Sperm Injection	
Abnormal SDF levels may adversely impact embryo development.	Zini (2011) Wdowiak et al. (2015) Alvarez Sedó et al. (2017) Zheng et al. (2018) Kim et al. (2019) Casanovas et al. (2019)
Abnormal SDF levels negatively affect IVF and ICSI pregnancy rates.	Osman et al. (2015) Oleszczuk et al. (2016) Simon et al. (2017) Nicopoullos et al. (2019)
The adverse effect of SDF on IVF/ICSI outcomes seems to be lower in ICSI studies than conventional IVF studies.	Li et al. (2006) Zini (2011) Simon et al. (2013) Zhao et al. (2014) Deng et al. (2019)
Abnormal SDF levels are associated with increased miscarriage risk in both IVF and ICSI studies.	Zini et al. (2008) Robinson et al. (2012) Zhao et al. (2014) Simon et al. (2017)
Testicular spermatozoa have lower SDF than epididymal and ejaculated spermatozoa.	Steele et al. (1999) O′Connell et al. (2002) Greco et al. (2005) Moskovtsev et al. (2010), Moskovtsev et al. (2012) Esteves, Sanchez‐Martin et al. 2015 Mehta et al. (2015) Hammoud et al. (2017) Xie et al. (2020)
Higher ICSI success rates are achieved with testicular spermatozoa than ejaculated spermatozoa in men with abnormal SDF levels.	Greco et al. (2005) Esteves, Sanchez‐Martin et al. et al. 2015, Esteves, Roque, et al. (2017) Bradley et al. (2016) Pabuccu et al. (2017) Arafa et al. (2018) Zhang et al. (2019) Herrero et al. (2019) Cheung et al. (2019) Esteves and Roque (2019) Xie et al. (2020)
8. Risk factors	
Environmental/occupational exposures have detrimental effects on SDF.	Sánchez‐Peña et al. (2004) Rubes et al. (2005) Evenson and Wixon (2005) Miranda‐Contreras et al. (2015) Lafuente et al. (2016) Jeng et al. (2016) Jamal et al. (2016) Radwan et al. (2016) Zhou et al. (2016) Zhu and Qiao (2015) Gandhi et al. (2017)
Cancer and exposure to chemotherapy and radiotherapy can increase SDF rates.	Bujan et al. (2014) Ståhl et al. (2006) Smit, van Casteren, et al. (2010) Marchlewska et al. (2016) Meseguer et al. (2008)
Tobacco and cannabis smoking have detrimental effects on sperm chromatin integrity and increase SDF rates.	Kumar et al. (2015) Cui et al. (2016) Sharma, Harlev, et al. (2016) Mostafa et al. (2018); Aboulmaouahib et al. (2018) Gunes et al. (2018) Boeri et al. (2019) Ranganathan et al. (2019) Verhaeghe et al. (2020)
Obesity may adversely affect SDF.	Morrison and Brannigan (2015) Sharma et al. (2017)
Men with advanced age have increased levels of SDF.	Simon et al. (2014) García‐Ferreyra et al. (2015) Rosiak‐Gill et al. (2019) Yatsenko and Turek (2018) Bertoncelli Tanaka et al. (2019) Evenson et al., 2020)
Lifestyle changes (e.g. avert smoking weight loss) may decrease SDF rates.	Faure et al. (2014) Rima et al. (2016) Jurewicz et al. (2018) Esteves, Santi, et al. (2020)

Abbreviations: SDF: sperm DNA fragmentation; IVF: in vitro fertilisation; ICSI: intracytoplasmic sperm injection

### 5.1 Varicocele

Varicocele represents the most frequent correctable cause of male infertility (Cho et al., 2016; Hamada et al., 2013). The testis responds to varicocele by producing excessive ROS, which can lead to SDF (Agarwal et al., 2012). Men with varicocele often have elevated OS markers and high SDF indices (Agarwal et al., 2012; Esteves, Gosálvez, et al., 2015; Roque & Esteves, 2018; Zini & Dohle, 2011). Approximately 50% of individuals with clinical varicoceles have elevated SDF. A 2018 systematic review including 21 studies and 1,270 infertile men showed that varicocele repair decreases SDF (Roque & Esteves, 2018). Studies evaluating pregnancy as an endpoint are few, but overall, they support the concept that couples who achieve pregnancy after varicocele repair have lower postoperative SDF rates than those who do not (Mohammed et al., 2015; Ni et al., 2014; Smit, Romijn, et al., 2010).

In general, there is a concurrent reduction of OS markers and SDF after varicocele repair (Roque & Esteves, 2018). A recent systematic review and meta‐analysis of nineteen varicocele studies including 1,153 men demonstrated that the pooled estimate for the mean difference (MD) in SDF values after varicocele repair (versus preoperative levels) was −8.3% (95% CI −10.3%, −6.4%; *p* < 0.0001; Roque et al., 2018). Elevated SDF rates have been reported in all grades of clinical varicocele, mainly grades 2 and 3 (Abdelbaki et al., 2017; Krishna Reddy et al., 2015; Ni et al., 2014, 2016; Sadek et al., 2011; Zaazaa et al., 2018).

Clinicians providing infertility care should consider advising men with clinical varicoceles of the association between SDF and OS. Before varicocele repair, SDF testing may be useful to detect and/or confirm a detrimental effect of varicocele on sperm quality and fertility, thus reinforcing the need for interventional therapy to reduce SDF and improve fertility. The added information provided by SDF tests can be notedly valuable when the decision to recommend varicocele repair is doubtful, in particular, for infertile men with (a) low‐grade (e.g. grade 1) varicocele and borderline to normal semen parameters (e.g. count, motility, morphology), and (b) moderate (grade 2) or large (grade 3) varicocele and semen parameters within normal ranges (Cho et al., 2017b).

After varicocele treatment, SDF retesting may be useful for monitoring the intervention′s outcome and guiding further management. The persistence of abnormal postoperative SDF values is a poor predictor for both natural and assisted conception. In such cases, couples should be counselled accordingly, and IVF‐ICSI offered, as discussed in the next sections. In contrast, the reduction of SDF is a good prognostic factor for conception both naturally and by ART (Roque & Esteves, 2018); the decision to pursue expectant management of ART will be based mainly on female factors.

On the other hand, the association between subclinical varicocele (i.e. nonpalpable on physical exam but vein dilation and reflux detected by colour doppler ultrasound ) and SDF remains equivocal. Although a controlled study involving 337 men reported a remarkable improvement in semen parameters and increased clinical pregnancy rates after repair of subclinical varicoceles (Cantoro et al., 2015), a 2016 systematic review and meta‐analysis compiling the data of seven randomised controlled trials (RCTs) demonstrated no improvement in pregnancy rates (OR 1.29, 95% CI: 0.99–1.67; Kim et al., 2016). However, none of the above studies assessed SDF rates. By contrast, two studies evaluated SDF in men with subclinical varicoceles. In a study involving 60 men, García‐Peiró et al. reported that while SDF rates were comparable between men with subclinical and clinical varicoceles, an improvement in sperm chromatin integrity post‐varicocelectomy was only seen in the subset of men with clinical varicoceles (García‐Peiró et al., 2014). In another study, Ni et al. also showed that routine semen parameters were lower in men with subclinical varicocele than in fertile men (i.e. normozoospermic healthy donors with at least one child) without varicocele; however, SDF values (assessed by SCSA) were neither statistically different between the groups nor did they change in a 6‐month follow‐up (Ni et al., 2016). Thus, more evidence is needed to allow any recommendation concerning the clinical value of SDF testing in men with subclinical varicocele (Majzoub, Agarwal, Cho, Esteves, 2017).

### 5.2 Unexplained and idiopathic infertility

Approximately 10%–30% of couples with infertility have no apparent clinical or laboratory alterations—using conventional diagnostic approaches—to explain their condition (Esteves et al., 2011; Hamada et al., 2012; Moghissi and Wallasch 1983). The term ‘unexplained infertility’ has been used when the basic investigations, including physical examination and tests for tubal patency, ovulation and semen analysis, are normal (Practice Committee of the American Society for Reproductive Medicine 2006; Esteves, Schattman, et al., 2015). The reported prevalence depends on the population studied and the criteria used for diagnosis. Despite some controversy regarding the optimal diagnostic panel for routine infertility evaluation (Practice Committee of the American Society for Reproductive Medicine 2012; 2015), the post‐coital test (PCT), or any other test of sperm–mucus interaction, has limited diagnostic value (Griffith & Grimes, 1990) and is no longer recommended for the routine evaluation of the infertile female. Along these lines, a routine semen analysis is also unable to identify sperm defects at the molecular level (Esteves, 2014; Esteves et al., 2012; Hamada et al., 2012).

Among men with unexplained infertility, elevated SDF rates are found in up to 20% of individuals (Esteves, Santi, et al., 2020; Feijó & Esteves, 2014; Gill et al., 2019; Oleszczuk et al., 2013; Saleh et al., 2003). Moreover, it has been shown that approximately 40%–50% of men with idiopathic infertility have elevated SDF rates (Aktan et al., 2013; Gill et al., 2019; Homa et al., 2019; Le et al., 2019; Simon et al., 2013). Men with idiopathic infertility have abnormal semen parameters—on a routine semen analysis—but no identifiable male factor (Agarwal et al., 2019; Darbandi et al., 2019; Esteves & Agarwal, 2011; Gunes & Esteves, 2020).

Overall, SDF rates are consistently higher in infertile men than presumed or confirmed fertile controls, irrespective of the assay used for measurement (Santi et al., 2018). The SDF results determined by TUNEL, SCD and SCSA have been independently related to the likelihood of achieving natural pregnancy, with lower SDF values associated with better reproductive success (Evenson et al., 1999; Evenson & Wixon, 2008; Malić Vončina et al., 2016; Spanò et al., 1998). For instance, the prospective ‘longitudinal investigation of fertility and the environment’ (LIFE) study provided level 1 evidence supporting an association between elevated SDF and a longer time to pregnancy (TTP; Buck Louis et al., 2014).

Thus, it may be prudent to offer SDF testing in couples with unexplained or idiopathic infertility, as an abnormal test result may indicate that damaged sperm chromatin might be the underlying infertility factor. In couples with unexplained/idiopathic infertility and elevated SDF, a reproductive urologist/andrologist evaluation is warranted to assess and possibly treat the underlying causes of elevated SDF. A decrease in SDF may allow these couples to achieve natural conception or eventually optimise the reproductive outcomes of assisted reproduction treatments. The use of ICSI may be a reasonable alternative in couples with no correctable factors for the male, particularly those with limited reproductive time window (e.g. advanced age, low ovarian reserve), as pregnancy rates by ICSI are less affected by elevated SDF than with the use of IUI and conventional IVF (discussed in next sections).

### 5.3 Recurrent pregnancy loss

Recurrent pregnancy loss is defined as two or more pregnancy losses from conception to 24 weeks of gestation (ESHRE Guideline Group on Recurrent Pregnancy Loss 2018). Current evidence indicates a plausible female factor‐independent relationship between RPL and SDF. In particular, miscarriage rates are increased in couples whose male partners have elevated SDF (Carlini et al., 2017; Evenson et al., 1999; Robinson et al., 2012; Zhao et al., 2014; Zidi‐Jrah et al., 2016). The studies by Robinson et al. and Zhao et al. compiled the data of couples undergoing IVF or ICSI, whereas Zidi‐Jrah et al. and Carlini et al. studied couples who had RPL after natural conception.

A systematic review and meta‐analysis of thirteen prospective studies showed that SDF rates were markedly higher in male partners of women with RPL than male partners of fertile control women (MD: 11.9%, 95% CI 4.9–18.8; McQueen et al., 2019). The pooled estimate was higher for TUNEL (14.2%, 95% CI 4.86–23.64) than SCD (3.5%, 95% CI −3.30–10.3), Comet (5.2%, 95% CI 0.31–10.1) and SCSA (10.1%, 95% CI 2.1–18.1). In another study, Tan et al. summarised the evidence of 14 RPL studies and found that SDF levels were higher in the affected couples compared with fertile controls (MD: 11.98%, 95% CI: 6.64–17.32, *p* < 0.001; Tan et al., 2019). In these studies, fertile controls were women with proven fertility with one or more live birth or ongoing pregnancy.

To date, the exact mechanism(s) involved in RPL in couples with SDF is not known. However, it has been speculated that DNA fragmentation not repaired by the oocyte may contribute to poor blastocyst development, implantation failure and miscarriage (Tan et al., 2019). A proposed mechanism involves oxidative stress. In this scenario, genetic/epigenetic changes in the zygote and developing embryo consequent to increased oxidatively induced SDF could cause RPL (Venkatesh et al., 2011). Specifically, excessive ROS can promote harm by modifying bases, creating abasic sites, chromatin protein cross‐linking and DNA strand breaks (both single and double) depending on the oxidative attack (Gosálvez, Fernández, et al., 2015). For instance, excessive ROS may lead to the formation of oxidised base adducts (e.g. 8OHdG). The sperm enzyme OGG1 cleaves oxidised base adducts out of the DNA, which creates a relatively unstable abasic site more prone to fragmentation (Aitken, 2017a; Feng et al., 2003; Lopes et al., 1998). Subsequently, the oocyte BER system will attempt to replace these oxidised bases by nonoxidised bases to correct the alterations after fertilisation and before syngamy.

Animal and human studies have shown that the zygote will respond to sperm DNA damage through a nonapoptotic mechanism if DNA damage exceeds the oocyte repair capacity or DNA repair mechanisms do not function properly. This mechanism acts by slowing paternal DNA replication and possibly producing chromosomal rearrangements, ultimately leading to poor embryonic development, implantation failure and miscarriage (Fernández‐Gonzalez et al., 2008; Gawecka et al., 2013; Gosálvez, Fernández, et al., 2015; Marchetti & Wyrobek, 2005; Menezo et al., 2007).

The type of DNA damage (single or double) seems to modulate the final effect. Recent evidence suggests that the presence of double DNA strand breaks (DS‐DBs) is more lethal than single‐DNA strand breaks and potentially associated with RPL, implantation failure and spontaneous miscarriage after IVF or ICSI (Ribas‐Maynou & Benet, 2019). By contrast, single‐stranded DNA breaks (SS‐DBs) seem to be more often associated with infertility and longer time to pregnancy in natural conception. The oxidised base adduct 8‐OHdG has been used as a marker to demonstrate that oxidative DNA damage is significantly elevated in spermatozoa of patients attending infertility clinics (De Iuliis et al., 2009). However, clinical trials are needed to establish the relationship between RPL with sperm DNA oxidation.

Like in unexplained infertility, SDF testing in couples with RPL may help identify the cases in which the damaged sperm chromatin contributes to the condition. This information would be useful for patient counselling and guide clinical management with the mindset of identifying potentially correctable underlying factors causing SDF. For example, a couple with RPL and normal bulk semen parameters found to have elevated SDF should have the male partner evaluated by a reproductive urologist/andrologist to rule out varicocele and other occult male factors. If no causative factor is identified, ICSI may be a reasonable alternative to overcome the problem.

### 5.4 Intrauterine insemination

In couples with unexplained infertility, IUI′s pregnancy rates decrease when SDF values (using the SCD assay) exceed 20% (Vandekerckhove et al., 2016). The likelihood of pregnancy success by IUI is also reduced (by 7.0‐ to 8.7‐fold) in the general infertile population when inseminations are carried out with samples from men with SDF levels >30% (measured by the SCSA in the neat semen; Bungum et al., 2004, 2007; Duran et al., 2002; Rilcheva et al., 2016).

Added to this, a systematic review and meta‐analysis compiling ten studies and over 2,800 IUI cycles demonstrated that SDF values ≥25% (measured by the SCSA or SCD assay) was associated with reduced pregnancy rates (10 studies; relative risk [RR]: 0.34, 95% CI 0.22–0.52, *p* < 0.001) and delivery rates (2 studies; RR 0.14, 95% CI:0.04–0.56, *p* < 0.001; Chen et al. 2019). These results have been confirmed in another meta‐analysis, including nine studies and 940 IUI cycles, which evaluated clinical pregnancy rates according to SDF results (RR: 3.15; 95% CI: 1.46–6.79; Sugihara et al., 2020).

On this basis, SDF testing may have value not only in couples experiencing unexplained IUI failures, but also those about to embark on this type of treatment. Elevated SDF will be indicative of poor prognosis with IUI. This information would be useful for patient counselling and also to guide clinical management. As discussed in previous sections, a reproductive urologist/andrologist should evaluate the male partner to rule out and fix any underlying male factors (e.g. varicocele, inadequate lifestyle), possibly causing SDF. After treatment, the patient might be retested to check if SDF was reduced to allow the continuation of IUI treatment. If no causative factor is identified, or elevated SDF persists after treatment, ICSI may be considered.

### 5.5 In vitro fertilisation/intracytoplasmic sperm injection

Most IVF/ICSI meta‐analyses concur that sperm DNA integrity impacts reproductive success. The studies of Li et al., Zini et al. and Zhao et al. showed that elevated SDF was associated with reduced pregnancy rates with conventional IVF but not ICSI (Li et al., 2006; Zhao et al., 2014; Zini, 2011). By contrast, Osman et al. and Simon et al. showed that elevated SDF adversely impacted both IVF and ICSI reproductive outcomes (Osman et al., 2015; Simon et al., 2017). The latter represents the most substantial data compilation to date. In their study, data from 70 studies, including over 17,000 IVF/ICSI cycles, were analysed, showing that elevated SDF was associated with reduced clinical pregnancy after either IVF (OR: 1.15, 95% CI: 1.05–1.27; *p* < 0.003) or ICSI (OR: 0.89, 95% CI: 0.80–0.99; *p* = 0.02; Simon et al., 2017). The miscarriage risk was also higher in couples undergoing IVF/ICSI with elevated (versus low) SDF rates (RR: 2.16; 95% CI: 1.54–3.03; *p* < 0.0001).

A meta‐analysis of 23 IVF/ICSI studies, including 6,771 cycles, corroborated these results (Deng et al., 2019). In this study, clinical pregnancy rates (23 studies; 6,771 cycles; RR: 1.57; 95% CI: 1.18, 2.09, *p* < 0.01) and miscarriage rates (25 studies; 3,992 patients; RR: 0.85, 95% CI: 0.75–0.96, *p* < 0.01) were negatively affected by the presence of elevated SDF; however, live birth rates were not apparently impacted (10 studies; 1,785 couples). Although the adverse impact of SDF on IVF and ICSI cycles has not been reported unequivocally (Cissen et al., 2016; Collins et al., 2008), an increasing body of evidence indicates that live birth rates decline in both IVF and ICSI patients when SDF rates (measured by Comet) exceeded the threshold levels (Nicopoullos et al., 2019).

The magnitude of effect size concerning the adverse effect of SDF on IVF and ICSI outcomes seems lower in ICSI studies than conventional IVF studies. The reasons are not fully understood, but a few possibilities to explain these observations have been raised by Lewis (Lewis, 2013). First, up to 30% of women having ICSI have no detectable problems. They may be fertile, and their oocytes can have more capacity to repair DNA damage even if the injected spermatozoon is of poor quality. This argument is supported by Meseguer and co‐workers (Meseguer et al., 2011), who showed that high‐quality oocytes from donors may offset the negative impact of sperm DNA damage on pregnancy. Secondly, in ICSI, the gametes are not subjected to prolonged culture; thus, spermatozoa may have less damage at the time of fertilisation than those exposed to incubation in culture media, as in IVF procedures.

In contrast to IVF, ICSI spermatozoa are injected into the oocyte within a few hours of ejaculation. This technical difference may protect them from laboratory‐induced damage; iatrogenic damage can occur when spermatozoa is maintained in vitro for long periods (Gosálvez, López‐Fernández, et al., 2011). Lastly, spermatozoa can be a source of ROS; if used in IVF, the oocyte may be exposed to oxidative assault during incubation. In ICSI, the oocyte is protected from this attack and can use its energies to repair the SDF immediately following fertilisation. Animal studies have shown intraspecies variation concerning sperm DNA resistance to damage under in vitro conditions, with an evident adverse impact of SDF on embryo development and pregnancy outcomes (Gosálvez et al., 2014; Johnston et al., 2016).

Increased miscarriage rates seem to be a common feature of IVF/ICSI cycles carried out with elevated SDF specimens. In a 2014 study compiling the data of 14 IVF/ICSI studies including 2,756 couples, Zhao and co‐workers showed that elevated SDF significantly impacted the likelihood of miscarriage (IVF/ICSI studies OR: 2.3; 95% CI: 1.55–3.35; *p* < 0.001; ICSI studies OR: 2.7; 95% CI: 1.4–5.1, *p* = 0.003; Zhao et al., 2014). These figures mean that if the average miscarriage rates are 10%–15%, they will reach 23% among couples subjected to IVF/ICSI with spermatozoa taken from semen specimens exhibiting elevated SDF. In practical terms, the net effect of SDF for a fertility centre performing 1,000 IVF/ICSI cycles per year with an average clinical pregnancy rate of 40% would be a reduction in approximately 80 pregnancies, ultimately resulting in a live birth rate reduction of up to 15%.

While the reasons for the reduced pregnancy rates among IVF/ICSI couples with elevated SDF are not entirely understood, genetic and epigenetic factors related to impaired sperm chromatin could explain suboptimal reproductive outcomes (Esteves & Agarwal, 2013; Esteves, Prudencio, et al., 2014; Mitchell et al., 2006; Strassburger et al., 2000). The DNA oxidative damage may cause mutations or dysregulate methylation processes and genetic pathways critical for embryo development and implantation (Aitken, 2017a; Dada, 2017; Feng et al., 2003).

Along these lines, a proposed mechanism to explain SDF‐related implantation failure after IVF/ICSI relates to deficiencies of the oocyte repair system to properly fix paternal DNA alterations (Champroux et al., 2016; Gosálvez, Fernández, et al., 2015). Both the oocyte repair capacity and the type and/or complexity of SDF vary from one cell to another, thus differentially affecting the embryo′s implantation potential. While both SS‐DBs and DS‐DBs can be repaired at the same DNA strand by direct ligation of 5′–3′ free ends, thereby evading the production of structural chromosomal abnormalities (Obe et al., 2002; van Gent et al., 2001), DS‐DNA breaks are more difficult to repair because there is no complementary strand to use as a template (Bernstein & Rothstein, 2009; Price & D′Andrea, 2013). Unrepaired DNA motifs may produce chromosomal rearrangements, which can generate high levels of genome instability. As a result, cell death and sudden embryonic loss may occur (Carrano & Heddle, 1973).

When DNA repair is complete, both the copy′s fidelity and the orthodox gene order housed in the chromosome allow the morula and blastocyst stages to be reached. In this case, the paternal genome would be normally regulated and expressed, and a successful pregnancy would ensue; otherwise, if the DNA repair processes were not wholly effective, implantation failure may occur. The latter seems to occur more often in association with DS‐DBs (Ribas‐Maynou & Benet, 2019).

The oocyte repair machinery modulates the adverse effect of elevated SDF on embryo development and pregnancy. However, oocytes of advanced age women are less efficient in repairing sperm DNA damage. The persistence of DNA breaks and mutagenic bases might ultimately increase the risk of embryo genetic and epigenetic defects (Aitken, 2017a; Champroux et al., 2016; Dada, 2017; Jin et al., 2015). Moreover, the SDF type (SS‐DBs or DS‐DBs) and quantity might also differentially affect embryo development. In studies using the Comet assay, it has been shown that DS‐DBs are more significant than SS‐DBs concerning embryo kinetics and implantation (Casanovas et al., 2019; Ribas‐Maynou & Benet, 2019).

Despite that, the data concerning the impact of SDF on embryo development remain ambiguous. In a 2011 systematic review compiling 3,226 IVF/ICSI cycles, elevated SDF was associated with impaired embryo development in 11 studies, whereas in 17 studies, the relationship was not evident (Zini et al., 2011). In oocyte donation programs, elevated SDF was shown to affect blastulation rates adversely, both in studies using the TUNEL assay (Alvarez Sedó et al., 2017) and the SCD test (Kim et al., 2019; Zheng et al., 2018), albeit not unequivocally (Antonouli et al., 2019). Furthermore, ICSI studies using time‐lapse technology demonstrated that the time to reach critical embryo development stages is negatively impacted by elevated SDF (by the alkaline Comet assay or SCD test; Casanovas et al., 2019; Wdowiak et al., 2015). Noteworthy, recent studies evaluating blastocyst ploidy indicate that SDF has no apparent adverse impact on embryo euploidy status (assessed by comprehensive 24‐chromosome genetic testing; Figueira et al., 2019; Gat et al., 2017).

Lastly, it has been suggested the health of infants could be impacted when natural or assisted inseminations are carried out with specimens with elevated SDF; the mechanisms are not fully understood, but the involvement of OS‐mediated altered expression of critical genes for sperm function, fertilisation and embryo development has been hypothesised (Aitken, 2017a; Rima et al., 2016; Vande Loock et al., 2012). Although the oocyte may tolerate oxidative sperm DNA damage in terms of fertilisation and pronucleus formation (Twigg et al., 1998), it is in the embryo′s subsequent development that the impact of oxidatively induced SDF seems to manifest more evidently (Burruel et al., 2013). This may relate to the presence of high levels of unresolved DNA damage leading to the induction of apoptosis or the creation of elevated mutational loads due to aberrant or defective DNA repair.

The hypothesis posed by Aitken (Aitken, 2017b) is that an oxidative attack on sperm DNA can lead to the formation of oxidative base adducts such as 8OHdG. In responding to such damage, spermatozoa can only rely on OGG1 enzyme in the base excision repair pathway (Smith et al., 2013). This glycosylase cleaves the oxidised base out of the DNA duplex to generate a corresponding abasic site that destabilises the ribose‐phosphate backbone leading to a β‐elimination or a ring‐opening reaction of the ribose unit and a consequential strand break. If this limited DNA repair pathway does not complete its task, 8OHdG residues persist in the spermatozoa and because the oocyte is poorly endowed with OGG1, they will be transferred to the zygote entering the S‐phase of the first mitotic division following fertilisation (Aitken et al., 2010; Smith et al., 2013). This phenomenon′s clinical significance is that 8OHdG residues are highly mutagenic, potentially causing an increase in the mutational load carried by the embryo (Aitken, 2017a), particularly, but not exclusively GC‐AT transversions (Ohno et al., 2014).

Similarly, oxidative stress in the germline can result in the formation of lipid aldehyde adducts on DNA involving compounds such as 4‐hydroxynonenal and 4‐hydroxyhexenal, both of which are also powerfully immunogenic (Feng et al., 2003). They could be responsible for increasing the mutation and epimutation loads carried by the offspring (Tharmalingam et al., 2017). Additionally, since the spermatozoon′s centromeres are responsible for orchestrating all cell division in the embryo, it is also possible that oxidative damage to this subcellular structure results in an impairment of ordered mitosis. Thus, deletions or sequence errors may be introduced into the developing embryo because of partial oocyte repair, and the health of resulting offspring may be affected (e.g. epigenetic changes, genetic diseases, metabolic diseases, neurological conditions and cancer; reviewed by Aitken, 2017a; Champroux et al., 2016).

The observed increase in mutational load in children of advanced age fathers (Kong et al., 2012) is an example of the above mechanism‐in‐action, which resonates with the link between advanced paternal age, oxidative sperm DNA damage and a range of pathologies including dominant genetic diseases in the offspring, achondroplasia and neurodevelopmental disorders (e.g. autism, bipolar disease, schizophrenia; Aitken, 2013, 2017a).

Similarly, there seems to be a link between the high levels of oxidative DNA damage observed in male smokers′ spermatozoa and the increased risk of progeny cancer (Lee et al., 2009). It has been reported that 80% of the novo structural chromosome aberrations in humans are of paternal origin (Tomar et al., 1984).

Notwithstanding these observations, there is limited clinical data concerning the impact of SDF on offspring's health to date. Reassuringly, it appears that children conceived by IVF and ICSI in couples with SDF do not have adverse birth characteristics (Bungum et al., 2012).

Sperm DNA fragmentation testing may have value in couples experiencing unexplained IVF/ICSI failures and, equally importantly, before commencing treatment. The information provided by the SDF test may be useful for patient counselling and to guide clinical management. In couples with an elevated SDF test result, a reproductive urologist/andrologist should evaluate the male partner to rule out any occult male factors possibly associated with the high SDF rates.

In cases where no causative factor is identified, or elevated SDF persists after treatment, ICSI using testicular spermatozoa has been suggested as an effective way to overcome unexplained ICSI failures (Alharbi et al., 2019; Arafa et al., 2018; Bradley et al., 2016; Cheung et al., 2019; Esteves & Lewis, 2020; Esteves & Roque, 2019; Esteves, Roque, et al., 2017; Esteves, Sanchez‐Martin, et al., 2015; Greco et al., 2005; Herrero et al., 2019; Pabuccu et al., 2017; Xie et al., 2020; Zhang et al., 2019).

The reason explaining the higher reproductive success using testicular spermatozoa for ICSI instead of ejaculated in these cases is not entirely understood. However, it may relate to the lower SDF rates in testicular specimens than in ejaculated and epididymal counterparts and the fact that testicular spermatozoa have not been exposed to oxidative‐induced damage during transit across the reproductive tract (Esteves, Gosálvez, et al., 2015; Greco et al., 2005; Hammoud et al., 2017; Mehta et al., 2015; Moskovtsev et al., 2010, 2012; Muratori et al., 2015; O′Connell et al., 2002; Steele et al., 1999; Suganuma et al., 2005; Xie et al., 2020).

### 5.6 Infertility risk factors

Male infertility risk factors include lifestyle conditions (e.g. tobacco smoking, obesity, metabolic syndrome), varicocele, genital infections, advanced age and exposure to toxicants (e.g. environmental, licit or illicit drugs [e.g. cannabis consumption], radiation, chemotherapy).

A positive association between exposure to air pollutants toxicants (e.g. particulate matter, nitrogen oxides, sulphur oxides, ozone) and SDF has been documented (Lafuente et al., 2016; Radwan et al., 2016; Rubes et al., 2005). Environmental and occupational exposure (e.g. polycyclic aromatic hydrocarbons, ionising and nonionising radiation, pesticides, endocrine disruptors, lead) can also increase SDF rates (Evenson & Wixon, 2005; Gandhi et al., 2017; Jamal et al., 2016; Jeng et al., 2016; Miranda‐Contreras et al., 2015; Sánchez‐Peña et al., 2004; Zhou et al., 2016; Zhu & Qiao, 2015). Additionally, therapeutic exposure to chemotherapy and radiotherapy can promote SS‐DBs and DS‐DBs in human spermatozoa (Bujan et al., 2014; Smit, van Casteren, et al., 2010; Ståhl et al., 2006).

Among lifestyle factors, tobacco smoking has an adverse influence on sperm chromatin integrity (Aboulmaouahib et al., 2018; Boeri et al., 2019; Cui et al., 2016; Fraga et al., 1996; Gunes et al., 2018; Kumar et al., 2015; Mostafa et al., 2018; Ranganathan et al., 2019; Sharma, Harlev, et al., 2016). Cannabis consumption can also impair sperm DNA quality (Verhaeghe et al., 2020). Along these lines, obesity might also affect sperm DNA quality (Morrison & Brannigan, 2015), albeit the evidence is less compelling (Sharma et al., 2017). The likely mechanisms in such patients include excessive peripheral conversion of testosterone to oestrogen, causing hypogonadism, increased ROS levels and increased testicular temperature due to excessive suprapubic fat.

Sperm DNA fragmentation increases with paternal age, particularly among men aged 40 years and older (Evenson et al., 2020; Rosiak‐Gill et al., 2019; Simon et al., 2014). A 2020 large cohort study, including 25,445 men aged 21–80 years from approximately 200 North American and European infertility clinics, who had SDF testing by SCSA, demonstrated that SDF rates increase as a function of age, remarkably after the age of 41 (Evenson et al., 2020). The authors used a logistic regression model to estimate the probability of having elevated SDF by age factor alone. Accordingly, a 40‐year‐old and a 50‐year‐old man were found to have a 20% and 40% chance, respectively, of exhibiting pathological SDF (DFI ≥ 25%; Evenson et al., 2020). Advanced paternal age might lead to mismatch DNA repair, which seems to be related to deficient sperm quality control during spermatogenesis (Yatsenko & Turek, 2018). In turn, these effects may translate into increased SDF, single‐gene mutations, and abnormalities in sperm chromosomes, ultimately resulting in poorer reproductive outcomes than that achieved in younger counterparts (Bertoncelli Tanaka et al., 2019; García‐Ferreyra et al., 2015).

Clinical data concerning the effects of tobacco smoking cessation and avoiding exposure to ambient or occupational chemicals on male fertility are lacking. However, a few studies suggest that lifestyle changes could improve sperm DNA quality. In one study, the Prudent diet (consisted of a high intake of fruits, vegetables, whole grains, nuts, fish, low‐fat dairy products) was shown to help reduce SDF rates (SCSA: 15.2% ± 10.4 versus 17.9% ± 8.1; *p* < 0.05) compared with the Western diet (high intakes of processed food, red meat, high‐fat dairy, refined grains, high energy drinks and sweets; Jurewicz et al., 2018).

A 2016 prospective controlled study from India, involving 56 fathers of children with retinoblastoma and 50 age‐matched fertile controls (i.e. men with a healthy child born in the last 1 year), indicated that SDF rates may be decreased by adopting meditation and yoga‐based lifestyle (Rima et al., 2016). After 6 months of yoga and meditation practice, SDF values decreased (SCSA; 31.5% ± 6.7 versus 21.9% ± 9.4; *p* < 0.01). However, no data were provided concerning the effect of SDF reduction on fecundity and offspring health. Lastly, an uncontrolled cohort study of six obese men with unexplained infertility showed that a nutritionist‐led dietary program associated with exercise over a 3‐ to 8‐month period was able to help reduce SDF values; in this study, the couples achieved full‐term deliveries after the intervention (Faure et al., 2014).

The laboratory evidence of defective sperm chromatin can be useful for patients′ counselling concerning overall reproductive health. It may help implement lifestyle modifications in couples who seek fertility counselling and family planning, particularly in those with infertility risk factors. As in the clinical scenarios previously discussed, male evaluation by a reproductive urologist/andrologist is warranted to assess the coexistent causes of elevated SDF that may be treated. SDF testing may also be used to monitor the effectiveness of health improvement programs on sperm DNA quality. Among patients with high SDF in whom no intervention is available to improve DNA quality, the information provided by the test can help decide the best treatment, IUI or IVF/ICSI, when both options are available. As for infertile men of advanced age, SDF testing results would help counselling about the pros and cons of conception using high SDF specimens.

### 5.7 Sperm cryopreservation

Sperm DNA fragmentation rates in the semen of men with a diverse range of cancer types can be as high as or even higher than that of infertile men (Marchlewska et al., 2016; Meseguer et al., 2008). Although the adverse effect of cancer and related therapy on sperm quality is not universal (Ribeiro et al., 2008), sperm banking is the only reliable option for fertility preservation in reproductive‐aged men (Esteves, Lombardo, et al., 2020).

Specimens are typically collected by masturbation, and the semen is cryopreserved using slow or rapid freezing protocols. Such samples are used for IUI or ART after thawing to allow these patients to father biological children. Before cryopreservation, the semen sample is analysed, and the baseline sperm variables (e.g. count, motility, morphology) are used to determine the ideal number of specimens to bank and prognosticate the assisted conception modality required for future conception. Moreover, a frozen aliquot is thawed for sperm cryosurvival assessment, which also helps estimate the total motile sperm number available and advise about the prospects of assisted conception.

The cryopreservation process can harm semen quality as it can increase ROS production, leading to excessive OS (Mazzilli et al., 1995). While the negative effects of cryopreservation on conventional semen parameters have been well documented, controversy still surrounds its impact on DNA integrity. Conventional freezing and vitrification are the two most commonly utilised methods for cryopreservation. A 2019 systematic review and meta‐analysis of 13 RCTs compared conventional freezing and vitrification using 486 vitrified and 486 conventional cryopreserved sperm specimens; Li et al., 2019). The post‐thawed total sperm motility (weighted mean differences 6.98%; 95% CI: 2.94; 11.02%; *p* < 0.0001) and progressive motility (weighted mean differences 4.59; 95% CI: 0.78; 8.39; *p* = 0.02) was significantly higher following vitrification than conventional freezing. However, SDF (reported in four studies) was not significantly affected.

Nonetheless, assessing SDF levels could help implement techniques to reduce the cryopreservation process′s hazards on the most vulnerable specimens. Utilising vitrification instead of conventional freezing methods is one example. Another example is the addition of antioxidant or antimutagenic compounds to sperm preparation media before cryopreservation; these substances may reduce OS‐induced SDF and improve post‐thaw motility and viability (Donnelly et al., 2000; O′Neill et al., 2019; Thomson et al., 2009).

Sperm DNA fragmentation testing before sperm banking may provide complementary information for some patients about semen quality. This information could help (a) select the optimal method for sperm freezing and (b) guide the later decision to use IUI or IVF/ICSI if both options are available.

## 6 RECOMMENDATIONS

**Figure 7:**
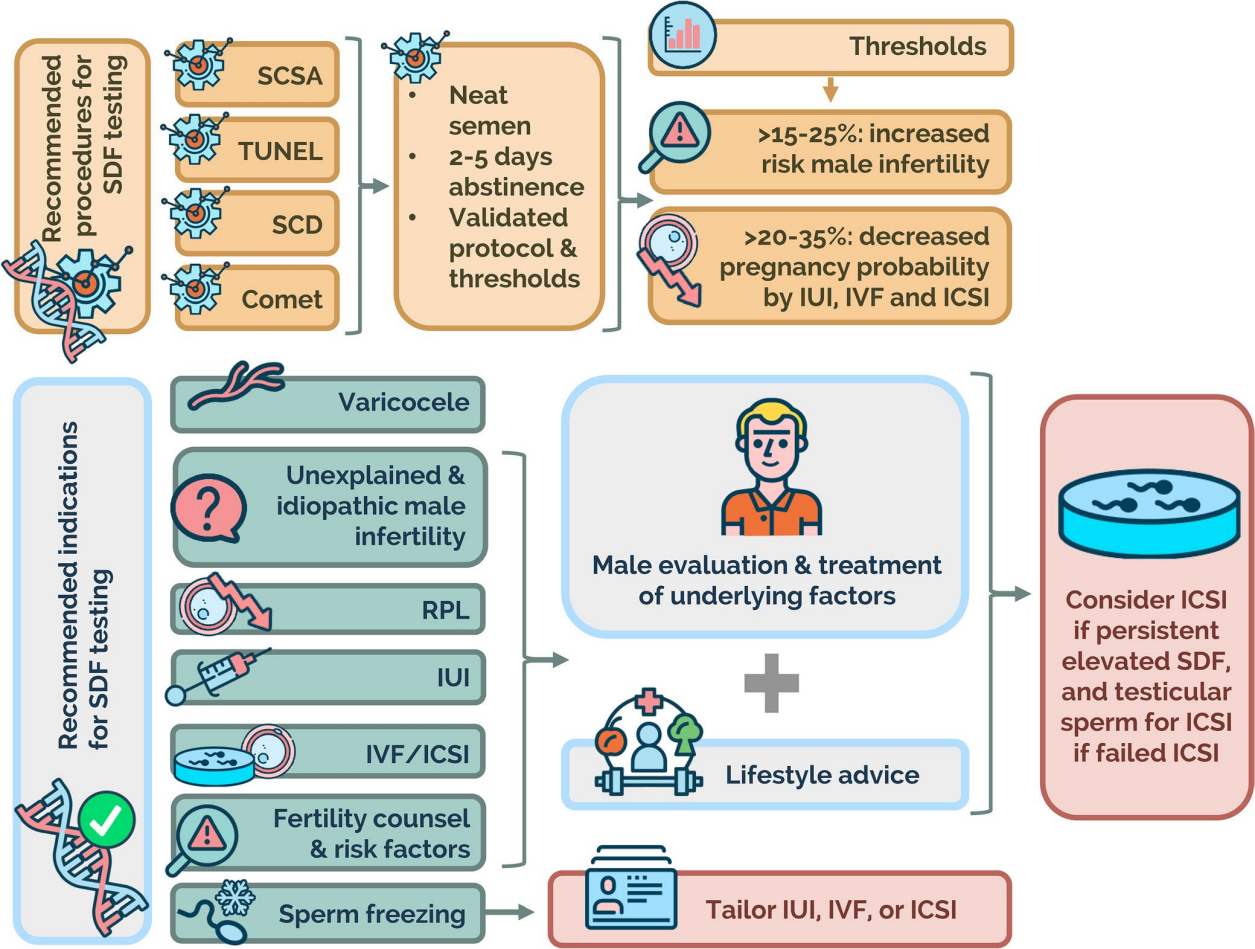
Pictorial summary of the recommendations for sperm DNA fragmentation testing and possible management in couples with elevated sperm DNA fragmentation. IUI, intrauterine insemination; IVF, in vitro fertilisation; ICSI, intracytoplasmic sperm injection; RPL, recurrent pregnancy loss

In this section, we provide recommendations concerning the technical aspects, indications and interpretation of SDF testing based on the evidence that has been identified, collated and analysed (see the summary of evidence in Tables 2 and 3). Also, recommendations are given regarding possible treatments to overcome infertility related to impaired sperm DNA quality. Tables 4 and 5 include all recommendations and grade their quality, as previously explained. A summary of how SDF testing should be conducted, interpreted and indicated, and the possible clinical management in the face of abnormal results is provided in Figure 7.

**Table 4:** Recommendations on technical aspects of Sperm DNA Fragmentation testing, clinical thresholds and interpretation of results

Recommendation	GDG strength rating^a^	OCEBM^b^ recommendation grade based on levels of evidence
The most reliable tests for assessing SDF are SCSA, alkaline Comet, SCD and TUNEL.	Conditional	Grade B
Any of the four SDF tests (SCSA, alkaline Comet, SCD and TUNEL) may provide valid information concerning the probability of reproductive success for couples embarking on IUI, IVF and ICSI.	Conditional	Grade B
A standardised protocol with strict quality control is essential for a reliable SDF testing result. Tests should be validated by the laboratory, with thresholds established based on the evaluation of fertile and infertile populations.	Strong	Grade A‐B
A neat semen sample should be used for SDF testing, collected after ejaculatory abstinence of 2–5 days.	Strong	Grade B
Patients should be asked not to have prolonged abstinence periods before the ejaculation that precedes the one used for testing.	Conditional	Grade D
A fixed ejaculatory abstinence length should be used for SDF testing when monitoring the effects of medical and surgical interventions aimed at decreasing SDF levels.	Conditional	Grade B
Fresh or frozen‐thawed specimens can be used for testing, but the analysis should start as quickly as possible after liquefaction (e.g. 30–60 min) or thawing.	Strong	Grade C‐D
If a frozen specimen is to be used for SDF testing, freezing should be immediately done after liquefaction is achieved.	Strong	Grade C‐D
Overall, thresholds of ~20% (SCSA, TUNEL and SCD), and 26% (alkaline Comet), best discriminate fertile from infertile men.	Conditional	Grade B
Overall, thresholds exceeding 20%–30% (SCSA, alkaline Comet and SCD) indicate a statistical probability of increased time to achieve natural pregnancy, increased miscarriage risk (after both natural and assisted conception), and low odds of reproductive success by IUI, IVF and ICSI.	Conditional	Grade B
SDF results—in combination with the current tools for infertility diagnosis—provide useful information concerning the probability of reproductive success.	Conditional	Grade B
SDF tests cannot perfectly discriminate fertile from infertile men or couples that will have a successful IUI, IVF or ICSI cycle from those that will not.	Strong	Grade B
The usefulness of any test for one partner is also dependent on the fertility of the other partner. Before testing, clinicians should have some understanding of the characteristics of SDF assays (e.g. sensitivity and specificity, positive and negative predictive value).	Strong	Grade B

Abbreviations: SDF: sperm DNA fragmentation; ICSI: intracytoplasmic sperm injection; IUI: intrauterine insemination; IVF: in vitro fertilisation; SCSA: sperm chromatin structure assay; SCD: sperm chromatin dispersion; TUNEL: Terminal deoxynucleotidyl transferase‐mediated dUTP‐biotin nick end labelling.

Grades of recommendations according to quality of evidence:

Grade A: consistent level 1 studies; Grade B: consistent level 2 or 3 studies or extrapolations from level 1 studies; Grade C: level 4 studies or extrapolations from level 2 or 3 studies; Grade D: level 5 or troubling inconsistent or inconclusive studies of any level.

Level 1 studies: systematic reviews with homogeneity of randomised controlled trials (RCTs) or level 1 diagnostic studies (1a); individual RCT with narrow confidence interval or validating cohort studies with good reference standards (2b).

Level 2 studies: systematic reviews with homogeneity of cohort studies or diagnostic studies (2a); individual cohort study or low quality RCT (2b), exploratory cohort study with good reference standards (2b).

Level 3: systematic reviews of case–control studies or moderate quality diagnostic studies (3a), individual case–control studies or nonconsecutive diagnostic studies (3b).

Level 4: case‐series or poor cohort/case–control studies or case–control diagnostic study.

Level 5: Expert opinion

http://www.cebm.net/oxford‐centre‐evidence‐based‐medicine‐levels‐evidence‐march‐2009/). Accessed June 7th, 2020.

^a^ Guideline development group (GDG) expert judgment; Strong recommendations imply that most individuals in that situation should receive the testing or intervention. Conditional recommendations imply that different choices might be appropriate for individual patients and that clinicians should help each patient reach a decision consistent with a patient‐centred approach.

^b^ Oxford Centre for Evidence‐Based Medicine Levels of Evidence (OCEBM Levels of Evidence Working Group)

**Table 5:** Recommendations on indications for sperm DNA fragmentation testing

Recommendation	GDG strength rating^a^	OCEBM^b^ recommendation grade based on levels of evidence
*Varicocele*		
Men with varicocele seeking fertility should be informed that varicocele may cause SDF and that repairing a clinical varicocele may alleviate SDF, potentially increasing the likelihood of reproductive success.	Strong	Grade B‐C
SDF testing may help identify patients with a profile that would not fit the standard indication of varicocele repair (e.g. clinical varicocele of any grade and normal/borderline routine semen analysis) but that can benefit from varicocele repair.	Conditional	Grade C
SDF testing may be used to monitor treatment outcomes.	Conditional	Grade C
SDF testing in subfertile men with subclinical varicocele is currently not recommended.	Strong	Grade C
*Unexplained infertility, idiopathic male infertility and recurrent pregnancy loss*		
Couples with unexplained infertility, idiopathic infertility and RPL should be informed that abnormal SDF levels may adversely impact their chances of achieving a live birth.	Strong	Grade B
SDF testing in couples with unexplained infertility, idiopathic infertility and RPL can be considered for explanatory purposes.	Strong	Grade B‐C
An abnormal SDF test result should prompt a complete male evaluation by a reproductive urologist/andrologist to help identify and possibly treat conditions associated with poor sperm DNA quality.	Strong	Grade D
ICSI may be considered if no correctable male factor is identified, or if abnormal SDF levels persist after treatment, particularly among couples with a limited reproductive time window.	Conditional	Grade B
*Intrauterine insemination*		
Infertile couples eligible for IUI treatment should be informed that abnormal SDF levels may adversely impact their chances of achieving a live birth.	Strong	Grade B
SDF testing may be considered before initiating IUI or after IUI failure.	Conditional	Grade B‐C
An abnormal SDF test result should prompt a complete male evaluation by a reproductive urologist/andrologist to help identify and possibly treat conditions associated with poor sperm DNA quality.	Strong	Grade D
Early ICSI may be considered in IUI eligible couples, or after failed IUI, if the male partner has high SDF levels, provided other measures to decrease SDF have been exhausted.	Conditional	Grade C
*In vitro fertilisation/intracytoplasmic sperm injection*		
Infertile couples eligible for conventional IVF treatment should be informed that abnormal SDF levels may adversely impact their chances of achieving a live birth.	Strong	Grade B
Infertile couples eligible for ICSI treatment should be informed that abnormal SDF levels may adversely impact their chances of achieving a live birth.	Conditional	Grade B
SDF testing may be considered before initiating IVF/ICSI or after unexplained failed IVF/ICSI.	Conditional	Grade B‐C
An abnormal SDF test result should prompt a complete male evaluation by a reproductive urologist/andrologist to help identify and possibly treat conditions associated with poor sperm DNA quality.	Strong	Grade D
ICSI rather than conventional IVF should be used to overcome infertility related to SDF.	Strong	Grade B
Among couples with ICSI failure and elevated SDF, testicular rather than ejaculated spermatozoa may be considered for sperm injection in subsequent treatment cycles.	Conditional	Grade B
The use of testicular spermatozoa in preference over ejaculated spermatozoa for ICSI, when both are available, may be particularly relevant for couples with no apparent reasons for a failed ICSI (e.g. no relevant female factors). This advice implies that a reproductive urologist/andrologist has evaluated the male partner and all possible corrective measures taken to improve overall reproductive health and sperm chromatin integrity.	Conditional	Grade D
*Fertility counselling for individuals with infertility risk factors*		
SDF testing may be considered to provide laboratory evidence of defective sperm chromatin to couples who seek fertility counselling and family planning, particularly when the male partner has an infertility risk factor.	Conditional	Grade C
Men with infertility risk factors (e.g. tobacco smoking, obesity, metabolic syndrome, exposure to environmental or occupational toxicants, use of licit or illicit drugs with gonadotoxic effects and advanced paternal age) should be informed that these factors may cause SDF and that lifestyle changes may alleviate SDF, potentially increasing the likelihood of reproductive success.	Conditional	Grade C
An abnormal SDF test result should prompt a complete male evaluation by a reproductive urologist/andrologist to help identify and possibly treat conditions associated with poor sperm DNA quality.	Strong	Grade D
An abnormal SDF test result may be used for counselling, reinforcing the importance of lifestyle changes and avoiding exposure to toxins.	Conditional	Grade C
Early ICSI may be considered for individuals with persistently high SDF levels despite corrective interventions, mainly when the reproductive window is limited.	Conditional	Grade D
The information provided by SDF testing may guide the choice of assisted conception modality, IUI, IVF or ICSI, in infertile couples with a male partner of advanced age.	Conditional	Grade D
SDF testing may be used to monitor the effects of lifestyle interventions.	Conditional	Grade D
*Sperm cryopreservation*		
SDF testing can be considered before sperm cryopreservation to provide additional information about semen quality.	Conditional	Grade D
The information provided by SDF testing may guide the decision to use IUI or IVF/ICSI for future conception with cryopreserved spermatozoa—in case both options are available—and the choice of the optimal sperm freezing method.	Conditional	Grade D

Abbreviations: SDF: sperm DNA fragmentation; RPL: recurrent pregnancy loss; ICSI: intracytoplasmic sperm injection; IUI: intrauterine insemination; IVF: in vitro fertilisation.

Grades of recommendations according to quality of evidence:

Grade A: consistent level 1 studies; Grade B: consistent level 2 or 3 studies or extrapolations from level 1 studies; Grade C: level 4 studies or extrapolations from level 2 or 3 studies; Grade D: level 5 or troubling inconsistent or inconclusive studies of any level.

Level 1 studies: systematic reviews with homogeneity of randomised controlled trials (RCTs) or level 1 diagnostic studies (1a); individual RCT with narrow confidence interval or validating cohort studies with good reference standards (2b).

Level 2 studies: systematic reviews with homogeneity of cohort studies or diagnostic studies (2a); individual cohort study or low quality RCT (2b), exploratory cohort study with good reference standards (2b).

Level 3: systematic reviews of case–control studies or moderate quality diagnostic studies (3a), individual case–control studies or nonconsecutive diagnostic studies (3b).

Level 4: case‐series or poor cohort/case–control studies or case–control diagnostic study.

Level 5: Expert opinion

http://www.cebm.net/oxford‐centre‐evidence‐based‐medicine‐levels‐evidence‐march‐2009/). Accessed 7 June 2020.

^a^ Guideline development group (GDG) expert judgment; Strong recommendations imply that most individuals in that situation should receive the testing or intervention. Conditional recommendations imply that different choices might be appropriate for individual patients and that clinicians should help each patient reach a decision consistent with a patient‐centred approach.

^b^ Oxford Centre for Evidence‐Based Medicine Levels of Evidence (OCEBM Levels of Evidence Working Group)

## 7 DISCUSSION

The primary goal of our CPG on SDF testing is to underscore the current indications of SDF testing based on best evidence and to help doctors counsel and explain the treatment options to patients with abnormal SDF. Our recommendations are based on evidence of varying quality, mainly moderate to low quality, like other male infertility guidelines. We acknowledge that the relevant literature lacks high‐quality studies in the field. However, this should not defer the effort to gather the best available evidence. Thus, the driving force of our collaborative effort was to translate the best evidence into current recommendations for standardised care while securing physician autonomy. We believe that the SDF testing shortcomings should not restrain healthcare providers from taking advantage of its clinical value, provided the information supporting that particular test for clinical decision‐making has been made clear to the patient.

Compared to previous guidelines, ours stand out for many reasons. First, we united not only reproductive urologists (SCE, AZ, RMC) with vast clinical experience but also a group of eminent scientists/andrologists (DE, JG, SEML, RKS) with seminal contributions to the development of the four major SDF tests (SCSA, Comet, SCD, and TUNEL). For the first time ever, the latter group worked together in a project of its kind, bringing readers the most detailed and practical information regarding SDF testing. Also, our guidelines included a distinguished reproductive endocrinologist (PH) with vast clinical experience, who added unique insights concerning the application of SDF testing in couples undergoing ART.

Second, we included several relevant studies in each subsection, strengthening the clinical utility of SDF testing and the recommendations made (Tables 2 and 3). Third, we expanded the SDF testing indications for a broader population of infertile men and couples undergoing IUI or IVF/ICSI. In varicocele, SDF testing can refine candidates′ selection for varicocele repair among the subset of infertile men with an equivocal indication for treatment. Additionally, SDF testing may helpful to the broad population of infertile men with clinical varicocele and abnormal semen parameters undergoing surgical repair. The information provided by the test can be used to monitor treatment effectiveness as a reduction in SDF rates post‐varicocele repair translates in a better reproductive success prognosis. After varicocele repair, men with persistently abnormal SDF values should be counselled accordingly as the time to natural pregnancy can be prolonged. For these men, ICSI rather than IUI or IVF should be considered if no other measures exist to alleviate SDF.

We also expanded the indication of SDF testing to all couples considering IUI or IVF/ICSI provided the minimum requirements for running an SDF test are met (see Table 1). The information provided by the test may help identify and guide management in couples that an elevated SDF could cause IUI or IVF/ICSI failure. In these cases, ICSI rather than IUI or conventional IVF should be recommended. This advice implies that a reproductive urologist/andrologist has evaluated the male partner and all possible corrective measures taken to improve overall reproductive health. In some cases, the reduction in SDF rates may help downgrade the assisted reproduction method, or even help achieve natural conception (reviewed by Esteves, Santi, et al., 2020).

Among couples with ICSI failure, our recommendation for testicular spermatozoa rather than ejaculated spermatozoa is overwhelmingly based on observational studies (reviewed by Esteves & Roque, 2019). Thus, caution should be exercised in this matter as sperm retrieval is not free of complications. Sperm retrieval and intracytoplasmic testicular sperm injections should be advocated in a considered manner preferentially to couples with ICSI failure after exhausting other resources to decrease SDF. When indicated, sperm retrieval should be carried out by a reproductive urologist/andrologist.

Lastly, we included new indications for testing, namely, fertility counselling and family planning, particularly among individuals with infertility risk factors and cancer patients who wish to bank spermatozoa for fertility preservation. The information provided by the SDF test may potentially offer guidance to family planners who are attempting natural conception, particularly among those with infertility risk factors and/or limited reproductive time window. The laboratory evidence of defective sperm chromatin should be used to counsel patients about the overall reproductive health and reinforce the importance of lifestyle modifications, including risk reduction. SDF testing can also be used to monitor patient compliance with health improvement interventions. The risks associated with a prolonged attempt to natural conception should be discussed with those individuals who remain with elevated SDF rates after interventions, and early ICSI may be considered.

Outstanding medical care delivery involves providing effective and safe care based on the best possible evidence. The foundations of evidence‐based medicine rely on the application of evidence that healthcare providers and patients can understand. Also, care provision should be driven by expert advice and patient‐shared decision‐making through meaningful conversations (Greenhalgh et al., 2014; Trost & Nehra, 2011). The literature concerning the clinical utility of SDF testing is increasing steadily, and in the future, the present CPG will undoubtedly need to be updated. CPG are evolving documents owing to the continued growth in medical knowledge. Hence, periodic review and update are of utmost importance to provide stakeholders with the most relevant practice guidance.

## 8 GAPS IN KNOWLEDGE AND RECOMMENDATIONS FOR FUTURE RESEARCH

Based on the published data and discussion of the available evidence, we identified various topics for which evidence is inconclusive or inexistent. The GDG recommends that future research focuses on the gaps in knowledge listed below.

- Establish which is the most informative SDF test for different clinical scenarios, and when a combination of tests is indicated.
- Develop a prognostic model for an individualised assessment of the chances of live birth and time to pregnancy according to SDF values and patient characteristics.
- Elucidate the exact mechanisms of oxidatively induced single‐strand and double‐strand sperm DNA breaks and further study their effects on reproductive outcomes.
- Perform epidemiological studies on the prevalence of elevated SDF among couples with unexplained infertility, RPL, males with idiopathic infertility, men with clinical and subclinical varicocele, couples undergoing IUI, IVF and ICSI, family planners with risk factors for infertility, and cancer patients at reproductive age who will bank spermatozoa.
- Study the relationship between implantation failure and RPL, and sperm DNA oxidation.
- Study the psychological impact of elevated SDF on men seeking fertility.
- Clarify the role of varicocele grade on SDF, and the role of varicocele repair to reduce SDF according to grade, the time needed for SDF improvement, and effects on reproductive outcomes.
- Study the effect of varicocele repair on reproductive outcomes in infertile men with clinical varicocele, elevated SDF and routine semen parameters within normal ranges.
- Study the association of SDF and subclinical varicocele and the effects of varicocele repair on reproductive outcomes.
- Study the impact of lifestyle interventions on sperm DNA quality and their impact on reproductive outcomes (preferable in prospective studies with appropriate controls).
- Clarify the role of antioxidant therapy for men with SDF.
- Compare the cost‐effectiveness of early ICSI versus expectant management in couples with unexplained infertility, RPL, family planners with infertility risk factors (preferably in prospective studies).
- Compare laboratory techniques to select spermatozoa with low DNA fragmentation for ICSI, in prospective trials involving couples with elevated SDF, controlled for age and other confounders.
- Compare the clinical efficacy of testicular spermatozoa for ICSI, in prospective randomised trials involving couples with elevated SDF, controlled for age and other confounders.
- Further research is needed on the clinical SDF thresholds to be used with each SDF test on IUI, IVF, and ICSI, using different endpoints (e.g. live birth, miscarriage).
- Clarify the role of oocyte quality on SDF repair.
- Establish the value of preimplantation genetic testing in couples with elevated SDF undergoing IVF/ICSI.
- Study the role of cryoprotectants and cryopreservation techniques to protect spermatozoa from DNA damage.

## 9 CONCLUSIONS

Male infertility is a common medical condition and a public health concern as it is associated with adverse effects on reproduction, overall health, reduced life expectancy and impaired quality of life. A comprehensive evaluation of male infertility can reveal severe and potentially life‐threatening underlying medical conditions. The prevention and management of male and female infertility are integral components of comprehensive sexual and reproductive health services needed to attain a sustainable development goal. Our CPG translates the best existing evidence into recommendations to provide the foundation for standardising care while maintaining clinicians′ autonomy. We herein reviewed the data supporting the indications of SDF testing in different infertility scenarios and elaborated recommendations based on best evidence and expert judgment.

## 10 TAKE‐HOME MESSAGES

- Infertility is a couple′s problem; thus, a single test of gamete dysfunction from just one partner is limited to predict the treatment outcome. However, SDF thresholds may reflect the probability of a successful reproductive outcome influenced by the SDF level and modulated primarily by females age.
- While SDF testing is not a replacement for the current tools for infertility diagnosis, it may add independent information about sperm quality, and its integration into fertility clinics may provide better counselling, diagnosis and treatment planning.
- Sperm DNA fragmentation testing in the clinic can help to:
  - Identify patients with potentially correctable underlying factors causing SDF, including the optimal selection of patients for varicocele repair.
  - Provide laboratory evidence of defective sperm chromatin to couples seeking fertility counselling and family planning, particularly when the male partner has infertility risk factors, as a way to counsel about fecundity prospects and reinforce the importance of lifestyle changes and avoid exposure to risk factors.
  - Better assess the semen quality of subfertile men of advanced paternal age for counselling and guiding clinical management.
  - Monitor the effects of interventions (e.g. varicocele repair, lifestyle changes);
  - Identify and guide management in couples where elevated SDF might contribute to unexplained/idiopathic infertility.
  - Identify and guide management in couples in whom elevated SDF might contribute to recurrent pregnancy loss and could cause IUI or IVF/ICSI failure.
  - Better assess the sperm quality of cancer patients who wish to bank sperm for fertility preservation to guide the choice of assisted conception modality optimally.
- The male partner of any infertile couple who is found to have elevated SDF should be evaluated by a reproductive urologist/andrologist to rule out varicocele and other occult male factors. The reduction in SDF rates may help the couple achieve natural conception, downgrade the assisted reproduction method and increase the likelihood of successful reproductive outcomes with IUI, IVF and ICSI.

## AUTHORS’ CONTRIBUTION

SCE coordinated the GDG and had a leading role in collecting the evidence, drafting the manuscript and handling the GDG’s comments. All participants contributed to the guideline development, discussed the key questions, synthetised the evidence, drafted recommendations and writing sections of the manuscript. All authors read and approved the submitted version.

## DISCLOSURE

SCE declares the receipt of unrestricted research grants and lecture fees from Merck outside the submitted work. PH reports receipt of unrestricted research grants from Merck, IBSA, Gedeon Richter, and MSD, and lecture fees from Merck, Gedeon Richter, MSD, and IBSA outside the submitted work. AZ declares shares in YAD‐Tech neutraceuticals. DPE is president director of SCSA Diagnostics, a company with a commercial interest in sperm DNA damage. SEML is an employee of Examenlab Ltd., a university spin‐out company with a commercial interest in sperm DNA damage. The remaining authors declare that the research was conducted in the absence of any commercial or financial relationships that could be construed as a potential conflict of interest.

## Supporting information

Fig S1‐S5

## Data Availability

The authors confirm that the data supporting the findings of this study are available within the article [and/or] its Supplementary Materials.
