# Supplementary material for: Sperm DNA fragmentation testing: Summary evidence and clinical practice recommendations
Source: Andrologia. 2020 Oct 27;53(2):e13874. doi: 10.1111/and.13874 (PMC7988559; doi:10.1111/and.13874)
Supplement: Supplementary file 1 — Fig S1‐S5 [file AND-53-e13874-s001.pdf]

SCSA® Diagnostics, Inc.
302 6th Street West - Suite B
Brookings, SD 57006
605 592-9071 / 866-219-1338
CLIA Certified: 43D0975076

# CLINICAL REPORT: Sperm Chromatin Structure Assay (SCSA)

**Patient:**

**Physician:**

**Date of Birth:**

**Clinic:**

**Date of Report:**

**Clinic City, State:**

**Collection Date:**

**DNA Fragmentation Index (%DFI; % sperm cells containing measurable DNA damage)**

a. ≤ 15% DFI = Excellent to Good Sperm DNA integrity
b. > 15% to < 25% DFI = Good to Fair Sperm DNA integrity
c. ≥ 25% to < 40% DFI = Fair to Poor Sperm DNA integrity
d. ≥ 40% DFI = Very Poor Sperm DNA integrity

**Note:** The above values relate to natural and IUI conceptions. When % DFI is above 25%, current literature suggests that the patient either try to reduce that number by medical intervention or change of lifestyle, or skipping IUI and go on to IVF/ICSI for greatest success. (www.scsatest.com for details).

**Hypothesis:** A high ratio of moderate DFI to high DFI sperm may be the most negative since Moderate DFI sperm have normal nuclear morphology and will likely fertilize but may have DNA damage beyond the repair capacity of eggs.

**High DNA stainability (HDS):** % sperm with immature chromatin and abnormal proteins; levels in the > 25% range are considered negative for pregnancy outcome.

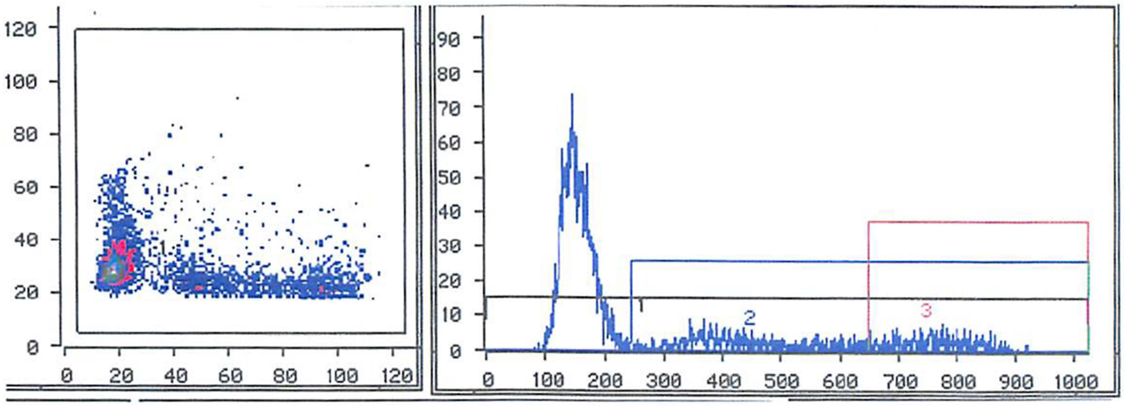


**DNA Fragmentation Index**

| File | % Total | % Moderate | % High | % HDS | Mean DFI | Std Dev DFI |
|---|---|---|---|---|---|---|
| 37 | 28.86 | 17.44 | 11.42 | 5.10 | 275.70 | 212.50 |
| 38 | 28.93 | 18.54 | 10.39 | 4.90 | 266.40 | 203.52 |
| **mean** | **28.9** | **18.0** | **10.9** | **5.0** | **271.1** | **208.0** |
| **std dev** | **0.0** | **0.8** | **0.7** | **0.1** | **6.6** | **6.3** |

**SCSA Results**

**DFI** = 29% **HDS** = 5%

**Estimate of Overall Sperm DNA Integrity**

Fair to Poor Sperm DNA Integrity

Donald Evenson, Ph. D., HCLD
President and Director

Supplementary Figure 1. Sample report SCSA

Examen

| REFERRING DOCTOR | | PATIENT DETAILS | SAMPLE DETAILS | |
|---|---|---|---|---|
| Name: | | Name: | Sample code: | 20SR00019 |
| Clinic: | Examen | D.O.B: 01/01/2020 | Type: | Ejaculate |
| Clinic ID: | N/A | | Date analysed: | 16 June 2020 |
| Email: | | | Report date: | 16 June 2020 |

## DEFINITIONS

**Average Comet Score (ACS)** is the mean of all of the Comets scored
**Low Comet Score (LCS)** is the proportion of sperm with low DNA Damage
**High Comet Score (HCS)** is the proportion of sperm with high DNA Damage

## COMET PLOT

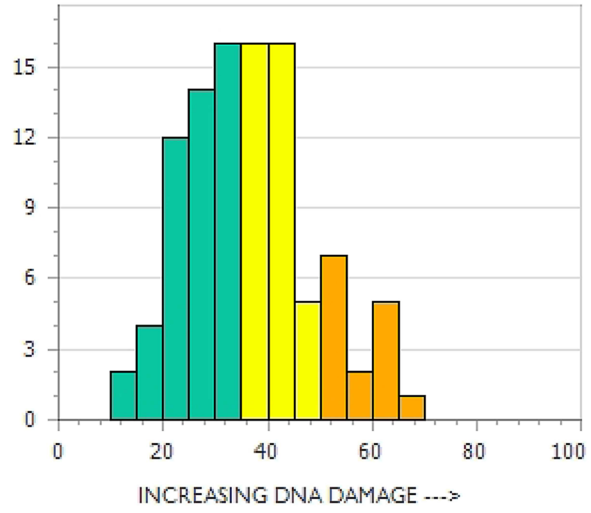


## REFERENCE PLOT: FERTILE POPULATION

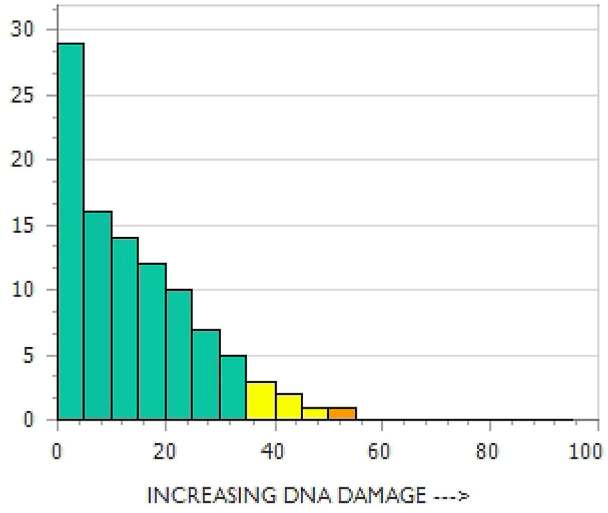


*Please note that the histograms are just a simple graphical description to aid understanding of results in comparison to fertile men.*

| | PATIENT RESULT | FERTILE RANGE | IVF RANGE | HIGH ICSI SUCCESS RANGE |
|---|---|---|---|---|
| Average Comet Score (ACS) | **37%** | 0-26% | 0-29% | 0-27% |
| Low Comet Score (LCS) | **41%** | 74-100% | 64-100% | 68-100% |
| High Comet Score (HCS) | **15%** | 0-4% | 0-6% | 0-10% |

## RESULT INTERPRETATION

**MALE INFERTILITY:** Outside the fertile range for all parameters indicating an increased risk of male infertility.

**IVF TREATMENT:** Outside the range for successful IVF for all parameters indicating ICSI as the optimal treatment

**ICSI SUCCESS RATE:** Outside the range for high ICSI success for all parameters. Improving DNA quality before treatment may increase the chance of a live birth.

Authorised by: *Professor Sheena Lewis PhD FRSB*

*UK State Registered Clinical Scientist*

Supplementary Figure 2. Sample report Comet assay

# Supplementary Figure 3. Sample report SCD test

Clinic
Andrology

**CLIENT CODE:**

**PATIENT NAME:**

**CLINIC CODE**

DATE OF SAMPLE RECEPTION
10/10/2020
DATE SAMPLE ANALYSIS
11/10/10/2020
DATE REPORT
12/10/2020

SAMPLE IDENTIFICATION CODE

REQUESTED INFORMATION
Assessment of Sperm DNA Fragmentation
(HaloSperm)

BASIC TECHNIQUE DESCRIPTION
Controlled sperm DNA denaturation and protein depletion producing expanding Haloes of dispersed chromatin around a core. Halo morphology is characterized. Large and Medium haloes of dispersion: NORMAL.

Small, no haloes or degraded: FRAGMENTED.

REFERENCE VALUES

1. Threshold for Infertility Diagnosis: 20-30%
   (Fertile range: 0-20%; Gray zone: 21-29%)
2. Threshold for IUI: 20% (normal range: 0-20%)
3. Threshold for IVF/ICSI: 25% (normal range: 0-25%)

PATIENT INFORMATION
SPERM DNA FRAGMENTATION: 69%

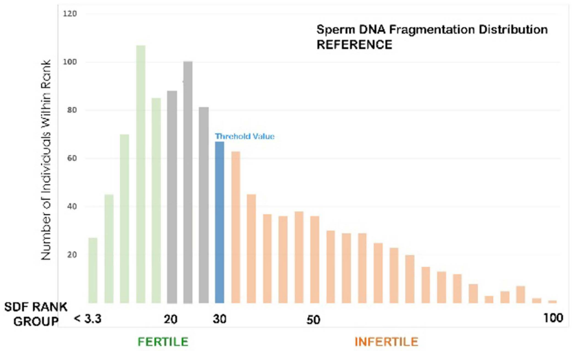


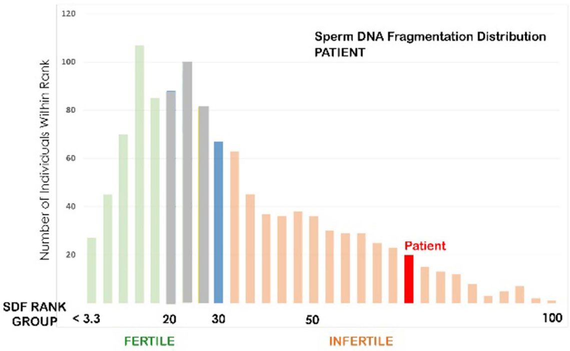


RECOMENDATIONS

**Male Infertility**: outside the fertile range (red) indicates an increased risk of male infertility.

**Intrauterine insemination**: Outside the range indicates a lower probability of success by IUI

**In vitro fertilization**: Outside the range indicates a lower probability of success by IVF, suggesting ICSI may be the optimal treatment.

ANDROFERT

ANDROLOGY LABORATORY
(ISO 9001:2015 CERTIFIED)

## General information

| ID | Name | Age | | Date |
|---|---|---|---|---|
| 07090 | | 27 | years | 12/06/2020 |

**Referring Doctor:** Dr. Sandro Esteves

**Clinical Diagnosis** Varicocele

| Specimen | Collection method | Collection site |
|---|---|---|
| Ejaculate | Masturbation | Collection room |

**Medication in use**

Vitamins

## Macroscopic analysis

| Color | Viscosity | Liquefaction | pH |
|---|---|---|---|
| Normal white | Normal | Complete | 8.0 |

**Abstinence lenght:** 2 day(s)

**Collection time** 09:36 am

**Analysis start** 40 minutes after collection

## Microscopic analysis

Reference values* (2020 WHO criteria)

| | Patient data | | No. cells analyzed | Std. error (%) | 5th percentile | 50th percentile | 95th percentile |
|---|---|---|---|---|---|---|---|
| Volume (mL) | 1.80 | | | | 1.5 | 3.7 | 6.8 |
| Count/mL (million) | 49.7 | | 398 | 5.0 | 15.0 | 73.0 | 213.0 |
| Total count (million/ejaculate) | 89.5 | | | | 39.0 | 255.0 | 802.0 |
| Motility (total; %) | 65 | % | 400 | 5.0 | 40% | 61% | 78% |
| Progressive motility (%) | 38 | % | | | 32% | 55% | 72% |
| Vitality (%) | NR | % | | | 58% | 79% | 91% |

*According to the European Society of Human Reproduction and Embryology, assessment of sperm viability should be carried out only when the percentage of immotile sperm exceeds 60%.

* References values from a fertile population, based in a study by the World Health Organization involving approximately 2,000 men who impregnanted their partners naturally in up to 12 months after stopping contraception. [1]
Sperm count carried out using the modified Neubauer chamber.
Glass slide and cover slip used for assessment of sperm motility (ambient temperature; 25oC).

## T.M.S.C.

| | | |
|---|---|---|
| Total motile sperm number de (million) | 34.0 | ≥7.20 (million) |

## Leukocytes (peroxidase test)

| | | | |
|---|---|---|---|
| Test result | Negative | | |
| No. peroxidase-negative round cells | 0.80 | million | |
| No. polymorphonuclear leukocytes* | NR | million | (normal < 1.0 million) |

* Reported only when the total number of round cells exceeds 1.0 million/ml.

## Sperm DNA Fragmentation (Sperm Chromatin Dispersion Test; SCD)

**Patient result**

Sperm with abnormal chromatin dispersion: **35** %

No. cells analyzed: 400

Std. error (%): 5.0

**Positive control:** 100 %

**Negative control:** 07 %

**REFERENCE VALUES:**

**1. Thresholds for Infertility Diagnosis: 30%**
**(Fertile range: 0-20%; Gray zone: 21-29%)**
**2. Thresholds for IUI: 20% (normal range: 0-20%)**
**3. Thresholds for IVF/ICSI: 25% (normal range: 0-25%)**

**Test method:**

Controlled sperm DNA denaturation and protein depletion producing expanding Haloes of dispersed chromatin around a core. Halo morphology is characterized. Large and Medium haloes of dispersion: NORMAL. Small, no haloes or degraded: FRAGMENTED.
The slides are stained and the percentages of sperm with nondispersed (abnormal) and dispersed (normal) chromatin loops are manually assessed by fluorescence or bright-field microscopy.

**Interpretation:**

**The Sperm DNA Fragmentation rate is** elevated **for the human ejaculate.**

**References**


1. WHO - World Health Organization laboratory manual for the Examination and processing of human semen. 5th. edition, 2010.
2. Esteves SC, Gosálvez J, López-Fernández C, et al. (2015) Diagnostic accuracy of sperm DNA degradation index (DDSi) as a potential noninvasive biomarker to identify men with varicocele-associated infertility. Int Urol Nephrol, 47, 1471-7.
3.Esteves SC, Sanchez-Martin F, Sanchez-Martin P, et. al. (2015). Comparison of reproductive outcome in oligozoospermic men with high sperm DNA fragmentation undergoing intracytoplasmic sperm injection with ejaculated and testicular sperm. Fertil Steril, 10, 1398-1405.
4. Esteves SC, Santi D, Simoni M. (2020). An update on clinical and surgical interventions to reduce sperm DNA fragmentation in infertile men. Andrology, 8, 53-81.


**Remarks**

None.

**Authorized by**

**Ellen Silva**

CRBM 34865

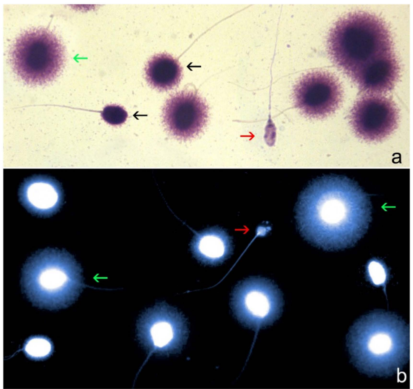


**Photograph legend: Nucleoids from human spermatozoa obtained with the improved SCD procedure (Halosperm, Halotech DNA, SL, Madrid, Spain) under bright-field microscopy and Wright's stain (a) and under fluorescence microscopy and DAPI staining (b). Green arrows indicate spermatozoa containing a normal DNA molecule. Yellow arrows indicate spermatozoa with a fragmented DNA molecule. Red arrows indicate a highly fragmented spermatozoon (degraded sperm).**

Supplementary Figure 4. Patient report SCD (Halosperm) test

Cleveland Clinic

# Andrology Center and Reproductive Tissue Bank

(ACCREDITED BY COLLEGE OF AMERICAN PATHOLOGISTS)

CLEVELAND CLINIC

## SPERM DNA FRAGMENTATION BY TUNEL ASSAY - REPORT

| REFERRING DOCTOR | PATIENT DETAILS | SAMPLE DETAIL |
|---|---|---|
| Name: Example | | MRN: 12345678 |
| Clinic: Hospital | D.O.B. 10/24/1985 | Type: Ejaculate |
| Email: lab@examplelab.com | | Date Analyzed: 04/19/20 |
| | | Report date: 04/20/20 |

| DEFINITIONS |
|---|
| Normal SDF: < 17% sperm staining positive by TUNEL using flow cytometry |
| High SDF: > 17% Percentage of sperm staining positive by TUNEL using flow cytometry |

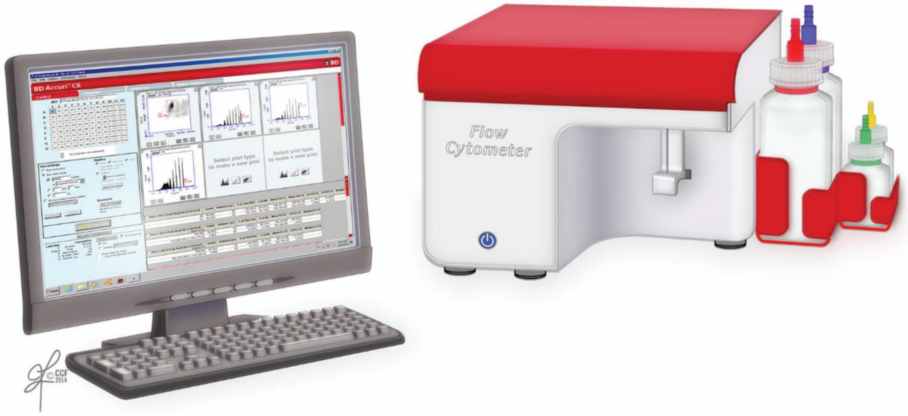


TUNEL assay using BD Accuri C6 flow cytometer

## PATIENT RESULT

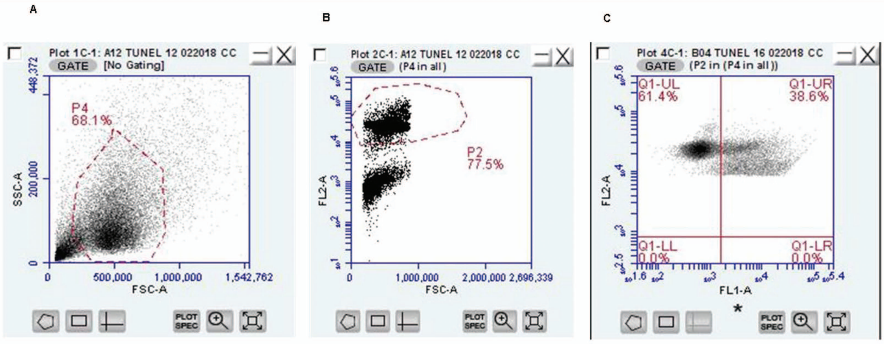


**A:** Gating of stained sperm **B:** PI positive cells **C:** Positive sample with 38.6% SDF

## RESULT INTERPRETATION

Result: 38.6% sperm DNA fragmentation (SDF), high DNA Fragmentation.

Reference values:

a) < 17 %: Good sperm DNA integrity

b) >17%: Poor sperm DNA integrity

MALE INFERTILITY: Sperm DNA fragmentation values >17% indicates an increased risk of male infertility.

DNA Fragmentation >20% may need IVF treatment.

DNA Fragmentation >36% may need ICSI treatment.

Authorized by: Ashok Agarwal Dr. Ashok Agarwal, PhD., HCLD

Director of Andrology Lab.

Supplementary Figure 5. Sample report TUNEL assay

©CCF 2020
